# Membrane Association Modes of Natural Anticancer Peptides: Mechanistic Details on Helicity, Orientation, and Surface Coverage

**DOI:** 10.3390/ijms22168613

**Published:** 2021-08-10

**Authors:** Mayra Quemé-Peña, Tünde Juhász, Gergely Kohut, Maria Ricci, Priyanka Singh, Imola Cs. Szigyártó, Zita I. Papp, Lívia Fülöp, Tamás Beke-Somfai

**Affiliations:** 1 Biomolecular Self-Assembly Research Group, Institute of Materials and Environmental Chemistry, Research Centre for Natural Sciences, Magyar Tudósok Körútja 2, H-1117 Budapest, Hungary; mayra.queme@ttk.hu (M.Q.-P.); kohut.gergely@ttk.mta.hu (G.K.); dr.mariaricci@gmail.com (M.R.); priyanka.singh@ttk.hu (P.S.); szigyarto.imola.csilla@ttk.hu (I.C.S.); 2 Hevesy György Ph.D. School of Chemistry, ELTE Eötvös Loránd University, Pázmány Péter Sétány 1/A, H-1117 Budapest, Hungary; 3 Department of Medical Chemistry, University of Szeged, Dóm tér 8, H-6720 Szeged, Hungary; papp.ibolya.zita@med.u-szeged.hu (Z.I.P.); fulop.livia@med.u-szeged.hu (L.F.)

**Keywords:** anticancer peptides, flow-linear dichroism, molecular dynamics, spectroscopy, peptide conformation

## Abstract

Anticancer peptides (ACPs) could potentially offer many advantages over other cancer therapies. ACPs often target cell membranes, where their surface mechanism is coupled to a conformational change into helical structures. However, details on their binding are still unclear, which would be crucial to reach progress in connecting structural aspects to ACP action and to therapeutic developments. Here we investigated natural helical ACPs, Lasioglossin LL-III, Macropin 1, Temporin-La, FK-16, and LL-37, on model liposomes, and also on extracellular vesicles (EVs), with an outer leaflet composition similar to cancer cells. The combined simulations and experiments identified three distinct binding modes to the membranes. Firstly, a highly helical structure, lying mainly on the membrane surface; secondly, a similar, yet only partially helical structure with disordered regions; and thirdly, a helical monomeric form with a non-inserted perpendicular orientation relative to the membrane surface. The latter allows large swings of the helix while the N-terminal is anchored to the headgroup region. These results indicate that subtle differences in sequence and charge can result in altered binding modes. The first two modes could be part of the well-known carpet model mechanism, whereas the newly identified third mode could be an intermediate state, existing prior to membrane insertion.

## 1. Introduction

Naturally occurring antimicrobial peptides (AMPs), or host-defence peptides (HDPs), represent one of the first evolved and successful forms of chemical defence for eukaryotic cells against bacteria, protozoa, fungi and viruses [1]. AMPs appear to have remained effective against specific pathogenic organisms over evolutionary time scales and have attracted increasing interest both in terms of their mechanisms of action and as a potential new source of designer molecules to address the growing threat of antimicrobial resistance. During the past decades, the research on antimicrobial peptides (AMPs) has revealed that some of them, termed anticancer peptides (ACPs), also show anticancer activity [2,3]. This is important, because according to the World Health Organization (WHO) [4], cancer remains a major cause of mortality, being the second leading cause of death for people above 70 years, in roughly half of the studied countries. These data point to insufficient current cancer treatments since most cancers cannot be permanently cured. Considerable progress has been achieved in respect to therapies [5], including chemotherapy, radiation, or hormone ablation [6,7]. However, severe side effects in normal cells and tissues caused by the insufficient specificity towards cancer cells are often obstacles to the clinical use of conventional therapeutics [5]. Therefore, novel treatment options with increased specificity are needed. ACP-based therapeutics have attracted great interest as drug candidates, offering many advantages due to their unique mechanisms of action, which can provide highly selective inhibition of tumour cell proliferation, migration, and tumour angiogenesis [6,8]. Over the past two decades, the number of ACPs entering the market or tested in clinical trials has been increasing [9,10]. In this respect, natural ACPs isolated from animals (including humans and amphibians), plants, and microorganisms are of great interest. In general, ACPs are short peptides, 5 to 40 residues in length, with high hydrophobicity and a net positive charge typically from +2 to +9. Electrostatic interactions between cationic ACPs and anionic cell membrane components are believed to be a key factor in their selectivity toward cancer cells. In addition, hydrophobic interactions also play important roles; thus, the delicate balance of the multiple effects could remarkably enhance the anticancer activity of ACPs [3,6]. 

However, despite the huge number of peptide sequences assumed to target lipid bilayers [11], and the extensive research on membrane-active AMPs and ACPs owing to the complexity of membrane-peptide systems, the exact molecular mechanistic details of how specific peptides associate with membranes and fulfil their function are still poorly understood. Further increasing the details is crucial to rationalizing ACP development processes and lowering the role of serendipity in identifying potent novel sequences. In general, ACPs can kill cancer cells mainly via cell membrane disruption mechanisms, similarly to how most AMPs exert function on microorganisms. Membrane disintegration by classical AMPs can occur through various general models, identified earlier and used throughout the field, such as pore formation in the lipid membrane (barrel stave and toroidal pore models), thinning of the membrane bilayer, membrane dissolution (carpet model), or lipid-peptide domain formation, membrane permeabilization, translocation across the membrane, and membrane-lysing [2,3,12,13,14,15,16,17]. Most of these functions are executed by the peptides in a helical secondary structure, and by now, biophysical studies have shown that many ACPs undergo a structural change from random to helical conformation upon being associated with a preferred lipid bilayer [2,18,19,20]. However, to reach control of desired membrane functionalities for future compounds and a better understanding of ACP membrane insertion, disruption mechanisms, interactions with associated surface proteins, oligomer formation preceding compromise of membrane integrity, etc., further structural details are needed.

**Scheme 1:**
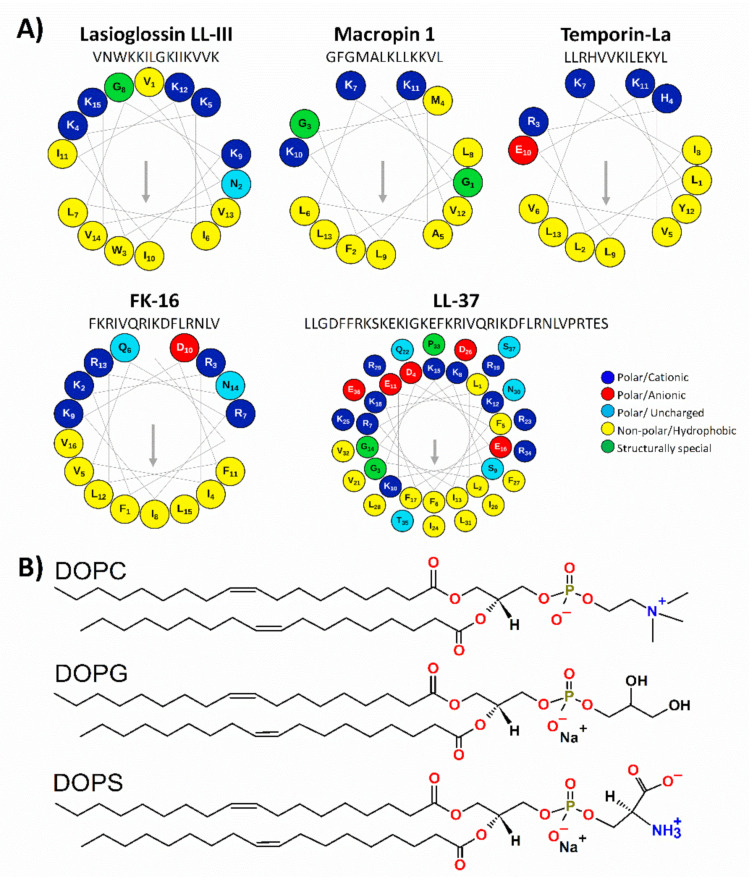
Chemical structures of the compounds used in the study. (**A**) Helical wheel diagram of the peptides was drawn with the software package Protein ORIGAMI [62]. The arrow indicates the hydrophobic face of the peptide. (**B**) model membranes built up of phosphatidylcholine (DOPC, PC), phosphatidylglycerol (DOPG, PG), and phosphatidylserine (DOPS, PS) were used throughout the study. Pure PC, PC/PS (80:20), and PC/PG (80:20) were used for mimicking spatial distribution of electrostatic features for neutral and negatively charged biomembranes, respectively. Note the zwitterionic but net neutral nature of PC, the single negative charge of PG, and the −/+/− charge distribution in the head-group of PS.

For progress here, we aimed to provide further subtle details beyond the formation of helical structures and set out to characterize the membrane-association mechanism of ACPs by employing combined theoretical and experimental biophysics, as well as improved model membranes. We selected five peptides (Table 1) that have demonstrated anticancer activity but for which mechanistic aspects have not been addressed yet in detail. To maintain a representative selection, those which are from natural sources and show membranolytic activities [2] were selected. At the same time, they differ in length, net charge, and origin (Scheme 1A). Lasioglossin LL-III [1,21,22,23,24] (or Lasio III) and the Macropin 1 [24,25,26] (or Macro1) are components of hymenopteran venom. Venoms of arthropods are a rich source of biologically active and pharmacologically interesting compounds [21]. Lasio III and Macro1 showed low or moderate haemolytic activity [24], and their effect on cancer cells has also been addressed (Table 2), but detailed information on their mechanism is lacking. Temporin-La [27,28,29] (or Tempo-La) is present in the skin of the frog *Rana temporaria*. Amphibians are the first group of multicellular organisms connecting water and land, and as a result, they developed excellent chemical defence systems composed of antimicrobial peptides [28]. Tempo-La was reported to inhibit the growth of various Gram-positive bacteria and cancer cells (Table 2) without damaging membranes of healthy mammalian cells [27]; however, the mechanism on how this peptide works remained to be elucidated. Finally, the human cathelicidin peptide, LL-37 [30,31,32,33,34], and its active fragment FK-16 are also involved [35,36]. LL-37 is a potent tumour suppressing peptide, and its ability to disrupt cell membranes has been demonstrated in several studies (Table 2). In this case, we use the full-length peptide as a reference to evaluate the potency of its fragment, as better activity against prokaryotes and nucleated cells was reported for FK-16 than for the precursor LL-37 [35,37,38].

Besides the selection of ACPs, it also had to be considered what peptide preferences towards cancer cells are related to the properties of the target membranes, such as membrane fluidity. Indeed, the membrane fluidity of cancer cells is greater than that of untransformed cells [39], which may enhance the lytic activity of ACPs by facilitating membrane destabilization. In contrast to normal mammalian cell membranes, which are mainly composed of neutral zwitterionic phospholipids, such as phosphatidylcholine (PC) [40], cancer cell membranes typically carry a net negative charge due to a higher than normal expression of anionic molecules. Among the latter, phosphatidylserine (PS) constitutes 3-9% of the total phospholipids of the membrane, and increasing evidence indicates that the exposure of PS on the outer leaflet could not only serve as a cancer cell marker [41] but also plays a key role in the selectivity of ACPs [42]. Hence, besides standard negatively charged vesicles, we employed PS in model membranes mimicking cancer cells (Scheme 1B). Moreover, to improve our understanding, extracellular vesicles (EVs), natively containing PS in the outer leaflet [43], were also exploited to study ACP action. EVs are lipid bilayer-enclosed vesicles produced by the human body that are extensively studied due to their important roles in intercellular communication [44], in diagnostics as biomarkers [45], and in therapeutic applications [46]. 

The combination of the above sets of compounds and approaches revealed novel details on membrane-associated helical ACPs, allowing us to allocate binding modes into categories. The structural and mechanistic insight provided here contributes to further understanding of the AMP/ACP mechanism of action.

**Table 1:** Properties of selected natural anticancer peptides used in the study.

Peptide	Family	Origin	Sequence ^a^	Length (aa)	Net Charge ^b^	<H> ^c^	<*μ_H_*> ^d^
**LASIO III**	Lasioglossins	*Lasioglossum laticeps* (hymenopteran venom)	VNWKKILGKIIKVVK	15	+6	0.54	0.77
**MACRO1**	Macropins	*Macropis fulvipes* (hymenopteran venom)	GFGMALKLLKKVL	13	+ 4	0.57	0.54
**TEMPO-LA**	Temporins	*Rana temporaria* (anuran skin)	LLRHVVKILEKYL	13	+3	0.49	0.73
**FK-16**	Cathelicidins	Derived from the peptide LL-37	FKRIVQRIKDFLRNLV	16	+5	0.32	0.78
**LL-37**	Cathelicidins	*Homo sapiens* (human)	LLGDFFRKSKEKIGKE**FKRIVQRIKDFLRNLV**PRTES ^e^	37	+6	0.23 ^f^	0.60 ^f^
						0.16 ^g^	0.73 ^g^
						0.27 ^h^	0.49 ^h^

**^a^** C-terminus of the peptides is amidated. **^b^** At neutral pH. **^c^** The mean hydrophobicity of each peptide was calculated as the average of hydrophobicities of each amino acid in the peptide chain assuming an α-helix and a segment of 11-residue window (average values), according to its octanol/water partition according to the scale of Fauchère, J., and Pliska, V [47], using the software HELIQUEST [48]. **^d^** The mean hydrophobic moment used to quantify the amphipathicity of the peptides was calculated according to the equation of Eisenberg et al. [49,50], assuming an α-helix and a segment of 11-residue window (average values), using the software HELIQUEST [48]. **^e^** The region corresponding to the fragment FK-16 is in bold. **^f^** Corresponding to the 1–11 residues of the sequence. **^g^** Corresponding to the 13–24 residues of the sequence. **^h^** Corresponding to the 25–36 residues of the sequence. (Additional information on these compounds can be found in the materials and methods section).

**Table 2:** Activity and proposed mechanism of selected natural anticancer peptides used in the study.

Peptide	Antibacterial Mechanism	Proposed Anticancer Mechanism	Cancer Cell Line ^a^	Haemolytic Activity ^b^ (LC50 [µM])	Reference
LASIO III	Outer and inner membrane permeabilization.	Cell membrane penetration and enter cells.	PC12, L1210, CCRF-CEM T, HL-60, HeLa S3, SW480	> 220	[21,22,23,24]
MACRO1	Membrane disruption and cell penetration. Permeabilization of the bacterial cell membrane.	-	CCRF-CEM, HeLa S3, SW480	~165	[22,24,25,26]
TEMPO-LA	Insertion into the plasma membrane.	Electrostatic interactions.	HeLa S3, SM MC7721, BEL-7402, A549, SW1116, HepG-2, BGC-823, HL-7702, HEK-293T	> 250	[2,27,28,29]
FK-16	Increased permeabilization of the membrane.	Induces cell death by both caspase-independent apoptosis and autophagy.	LoVo, HCT116, HT-29	~125 ^c^	[35,37,51,52,53]
LL-37	Targets anionic bacterial membranes via the carpet or toroidal pore model.	Induces apoptosis and depends mostly on the ability to act as a ligand for different membrane receptors whose expression varies on different cancer cells.	HT-29, HCT116, SW1116, SW620, SW480, LoVo, AGS, TMK1, Jurkat	> 70	[37,38,51,54,55,56,57,58,59,60,61]

**^a^** Cell lines: Pheochromocytoma of the rat adrenal medulla (PC12), mouse lymphocytic leukaemia (L1210), human lymphoblastic leukaemia (CCRF-CEM T), human promyelocytic leukaemia (HL-60), human cervix carcinoma (HeLa S3), human colon adenocarcinoma (SW480), human hepatocarcinoma (SM MC-7721), human lung adenocarcinoma epithelial (A549), human colorectal carcinoma (SW1116), human gastric carcinoma (BGC-823), human hepatocellular liver carcinoma (HepG2), human embryonic kidney (HEK 293T), human liver (HL-7702), human colon cancer (LoVo), (HCT116), (HT-29) and (SW620), human gastric adenocarcinoma (AGS), gastric adenocarcinoma (TMK1), human T leukaemia (Jurkat). **^b^** LC_50_ refers to the lytic concentration, i.e., the concentration at which 50% of erythrocytes are lysed. **^c^** Exhibited 13.61 ± 3.29% lysis at that concentration.

## 2. Results 

### 2.1. Peptide Structural Changes in the Presence of Lipid Membranes

**Figure 1:**
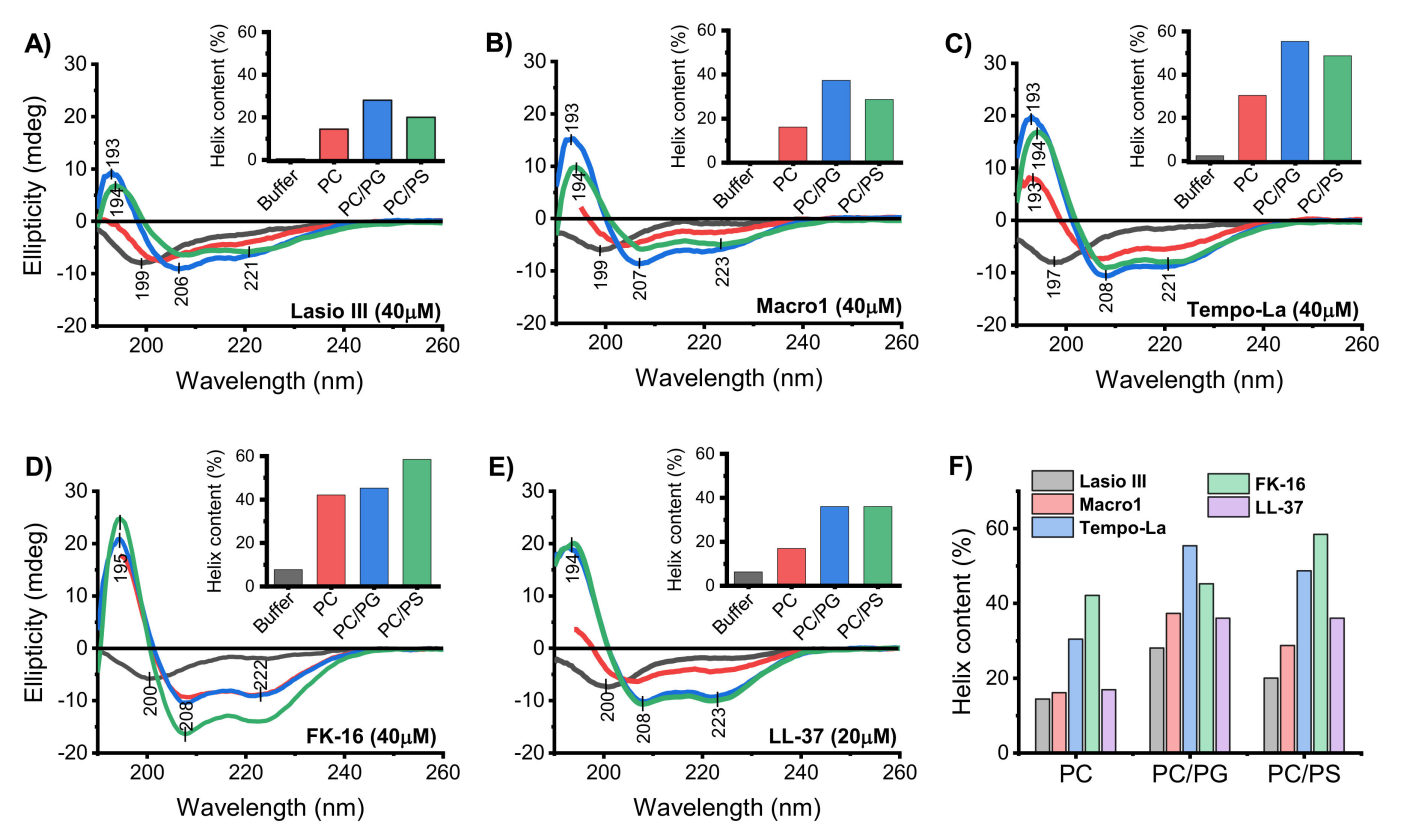
Peptide structural changes induced upon binding to model membranes assessed by CD spectroscopy. (**A**–**E**) Far-UV CD spectra in the presence and absence of the model membranes PC, PC/PG, and PC/PS. Spectra were collected at 635 µM for (**A**–**D**) and at 320 µM for (**E**) lipid concentration resulting in the same peptide-to-lipid ratio of 1:16. Insets show the calculated helix content. (**F**) Calculated peptide helix content with various lipid membranes. Helix content was calculated using the online tool (http://bestsel.elte.hu) [67].

Conformational variations were assessed first by means of CD spectroscopy, collecting spectra in the far-UV region with PBS buffer. The free peptides exhibited a single negative band centred at around 197–200 nm with no significant shoulder in the 210–230 nm region (Figure 1A–D), in accordance with their intrinsically disordered state. In the case of LL-37, the CD spectrum was recorded in low ionic strength Tris buffer with no added salt, as inorganic anions induce the α-helical conformation of the peptide (Figure 1E) [30,63,64]. The structural order of the studied ACPs was tested upon interaction with pure PC, PC/PG, and PC/PS liposomes, mimicking spatial distribution of electrostatic features for neutral and negatively charged biomembranes, respectively. Note that while other lipid components could also be considered here, that would, nevertheless, limit consistent comparisons with previous related results. Furthermore, some of the components, such as cholesterol, may have a marked effect on ACP behaviour; however, its content can drastically vary between different membranes or could even be missing, e.g., from bacterial bilayers. Thus, this consideration justifies the current choice of lipids and makes feasible the relative comparison of ACP properties as follows. In the presence of PC liposomes, the interaction with the lipid bilayer provoked the main negative band of the peptides to be red-shifted to 203–208 nm together with a positive band developing in the short-wavelength region at around 193-195 nm and a negative shoulder at 221–223 nm (Figure 1A–E). This is in line with partial helical conversion [65,66], which is observed most markedly for Tempo-La and FK-16 (Figure 1C,D). With PC/PG and PC/PS liposomes, all five peptides folded into a definite helical conformation (Figure 1A–E), where the spectral variations indicate longer, more regular helices in comparison with PC. This was also supported by the calculated helix content (Figure 1F, Insets Figure 1A–E). In general, the peptides showed a clear preference towards the negatively charged liposomes, particularly Tempo-La, FK-16 and LL-37. For Lasio III, Macro1, and Tempo-La, a somewhat higher helix content was estimated with the PC/PG liposomes (~40–50%) relative to the PC/PS liposomes (~30–40%). In contrast, the gain in helicity for FK-16 was highest with the PC/PS liposome (~60%), while the helicity was calculated to be the same (~43%) with PC and PC/PG. In the case of LL-37, the helix content was found to be the same (~40%) with the negatively charged bilayers. It is to be noted that the fragment FK-16 behaved differently compared to its parent LL-37, as FK-16 folded into a highly helical conformation with all model membranes used. Besides, out of the five peptides studied, Lasio III showed the lowest helicity content in the presence of liposomes for all lipid compositions studied. The conformational effect of the lipid bilayers on the peptides was also assessed by IR spectroscopy, revealing peculiar variations in dry film samples (Figure S1 and related text).

### 2.2. Position and Binding Depth of the Peptides in the Membrane/Interaction of the Peptides with Lipid Groups in the Membrane

**Figure 2:**
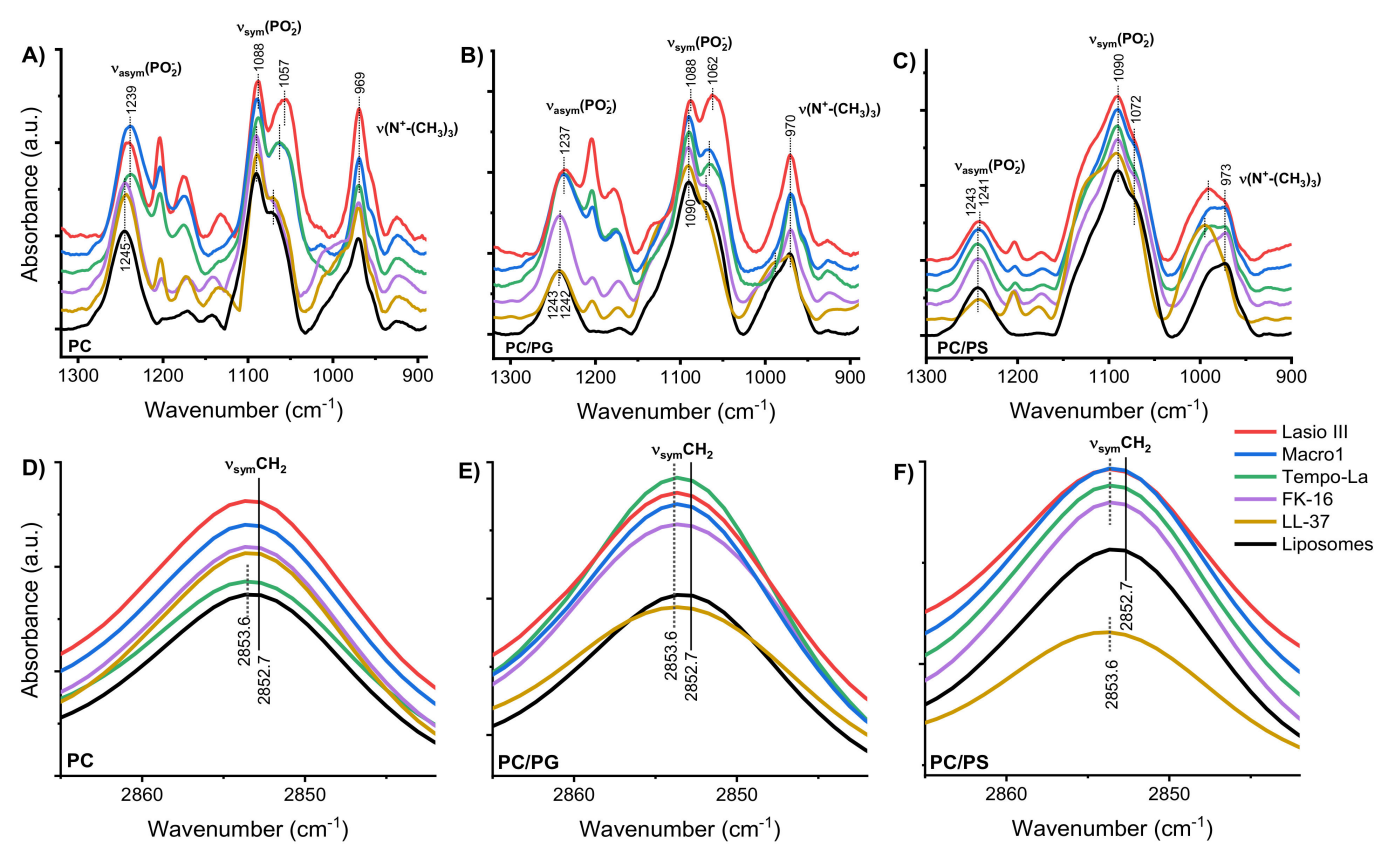
Membrane interaction studied by infrared spectroscopy (IR). Representative infrared spectra in the lipid head-group region of (**A**) PC, (**B**) PC/PG, and (**C**) PC/PS membranes alone and upon interaction with the studied peptides. The analysed vibrational modes, namely ν_asym_(PO_2^−^_), ν_sym_(PO_2^−^_), and ν(N^+^-(CH_3_)_3_), refers to the asymmetric and symmetric phosphate stretching, and to the asymmetric choline stretching, respectively. Representative infrared spectra of the acyl CH_2_ group region of (**D**) PC, (**E**) PC/PG, and (**F**) PC/PS membranes alone and upon interaction with the studied peptides. Peptide and lipid concentrations were 80 µM and 1.27 mM, respectively, for all the peptides except for LL-37 which was 40 µM and 0.635 mM, respectively, resulting in the same peptide-to-lipid ratio.

IR spectroscopy can be used to explore the conformational effect of the lipid bilayers on the peptides; nevertheless, this technique also allows one to monitor peptide-membrane interactions by focusing on the changes in the properties of the lipids [68,69,70,71]. The analysis of the spectral regions characteristic of phospholipid vibrations revealed alterations in the polar head-group moiety (~1200–950 cm^−1^, Figure 2A–C), the lipid ester neck (~1735 cm^−1^, Figure S1), and perturbations in the lipid order (~2850 cm^−1^, Figure 2D–F). 

The phosphate vibrations of zwitterionic PC liposomes were particularly perturbed by Macro1 and Tempo-La, which induced a shift of the ν_asym_PO_2^−^_ (Figure 2A). Moreover, increasing the intensity of the ν_sym_PO_2^−^_ at ~1060 cm^−1^ compared to the lipid alone (Figure 2A) for the peptides Lasio III, Macro1 and Tempo-La suggested an interaction with the phosphate groups and an impairment of the lipid head-group packing [72,73]. Regarding the anionic PC/PG liposome (Figure 2B), analogously to PC membranes, Lasio III, Macro1 and Tempo-La induced a similar shift in the ν_asym_PO_2^−^_ and an increased intensity in the ν_sym_PO_2^−^_, whereas a modest shift of ν_asym_PO_2^−^_ was induced by LL-37 and FK-16. A peculiar perturbation of ν_sym_PO_2^−^_ and choline stretching at ~970 cm^−1^ can be observed only in the case of the 37-mer peptide (Figure 2B). For PC/PS liposomes, a significant effect was revealed with Lasio III and LL-37, which caused alteration of the ν_asym_PO_2^−^_ and choline stretching vibrations (Figure 2C).

For the ester C=O stretching vibration (Figure S1B–D), a shift from 1737 to 1735 cm^−1^ in the presence of the peptides Lasio III and Tempo-La was detected with PC liposomes (Figure S1B), which is indicative of peptide binding down to the lipid neck region. In the case of anionic PC/PG and PC/PS liposomes (Figure S1C,D), a shift (from 1736 to 1737 cm^−1^) was detected for mostly all the studied peptides, especially for Lasio III and LL-37, which suggests structural arrangement/alterations in the polarity and/or hydration of the polar-apolar interfacial region of the lipid bilayer upon interaction with the ACPs.

Lastly, the symmetric stretching vibration of the methylene groups, ν_sym_CH_2_ at ~2850 cm^−1^, could be used for semi-quantitative characterization of concomitant changes in hydrocarbon chain conformational disorder [74,75]. In comparison with the pure lipid systems, for PC liposome, small shifts to higher wavenumbers and broadening can be detected upon addition of Tempo-La (Figure 2D). More pronounced shifts and peak broadening were detected with the negatively charged liposomes for the studied peptides (Figure 2E,F). The most remarkable changes were induced by LL-37, particularly with PC/PS, where a shift from 2852.7 cm^−1^ to 2853.6 cm^−1^ indicated more disordered acyl chain packing upon peptide binding (Figure 2F) [76].

### 2.3. Orientation of the Peptides in the Membrane

Linear dichroism (LD) spectroscopy was used to study the orientation and alignment (binding geometry) of the peptides bound to lipid bilayers. Linear dichroism gives information on the orientation of chromophores in a macroscopically aligned system as it measures the differential absorption of linearly polarized light parallel and perpendicular to an orientation axis [66,77]. Liposomes can be deformed by shear flow in a rotation Couette cell, resulting in slightly ellipsoidal vesicles which align in the flow [78]. Thus, the sign and magnitude of each peptide absorption band in the LD spectrum reports on the orientation of the corresponding transition moment relative to the membrane [78,79,80].

**Figure 3:**
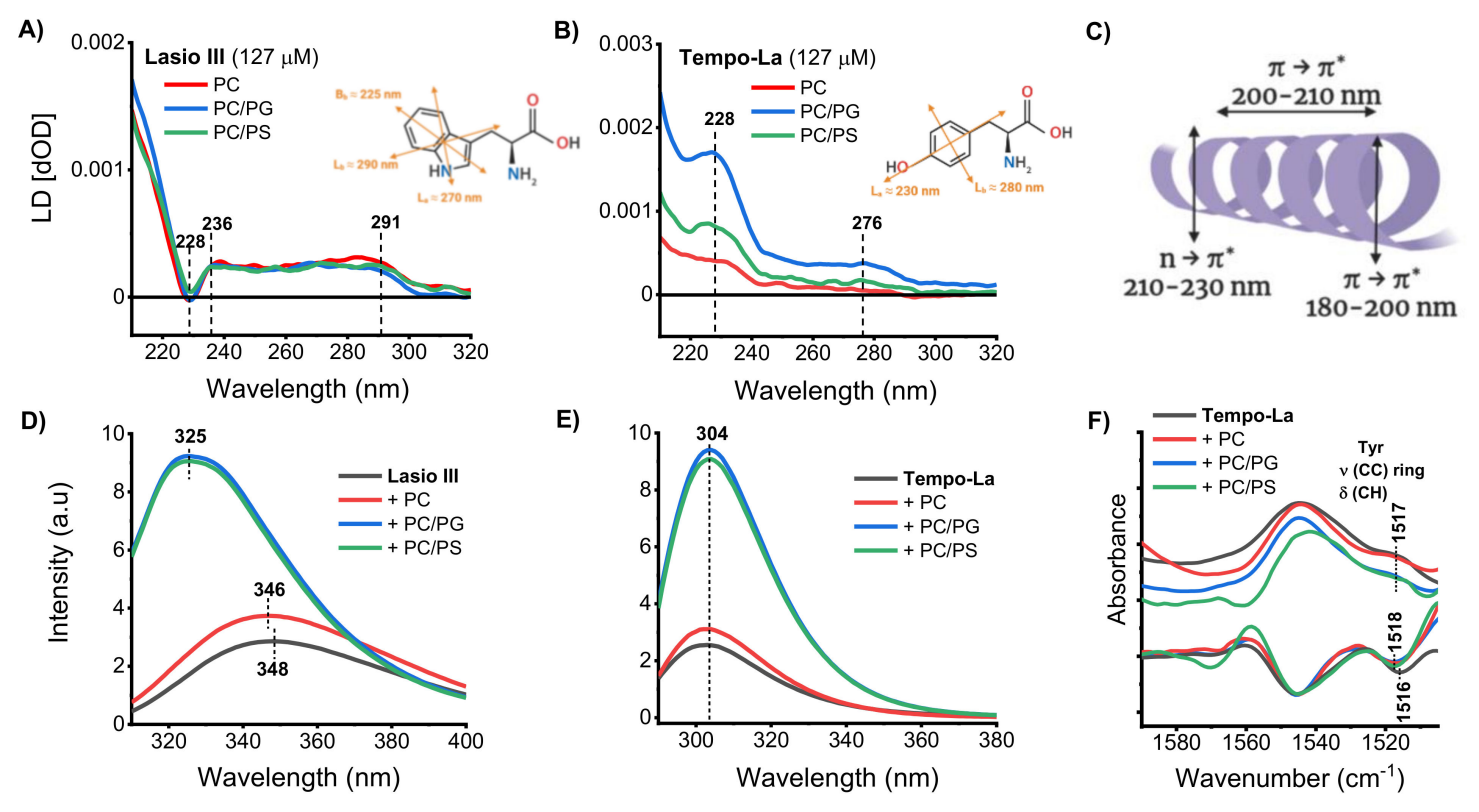
Peptide orientation upon binding to lipid membrane. (**A**,**B**) LD spectra of Lasio III (**A**) and Tempo-La (**B**) in the presence of the model membranes PC, PC/PG, and PC/PS. Spectra were collected at 1.27 mM lipid concentration. (**C**) Schematic representation of the transitions moment directions of an α-helical peptide secondary structure. (**D**,**E**) Fluorescence emission spectra of Lasio III (Trp fluorophore, excited at 295 nm) (**D**) and Tempo-La (Tyr fluorophore, excited at 275 nm) (**E**) in the presence and absence of model membranes. Peptide and lipid concentrations were 2 and 100 µM, respectively. (**F**) IR analysis of the tyrosine band of Tempo-La alone and in the presence of lipid membranes, showing the Tyr vibration band region. Second derivative is shown below and was used for peak identification. Peptide and lipid concentrations were 80 µM and 1.27 mM, respectively.

In the presence of model membranes, all five peptides displayed a positive peak at ~210 nm (Figure 3A,B and Figure S2), which corresponds to the low-energy component of the peptide bond $\pi \to \pi^{*}$ transition moment along the helix axis (Figure 3C) [78,81,82,83]. Thus, the positive sign of this peak suggests that the helical part of the peptide lies parallel to the surface of the membrane. Exceptionally, the Tempo-La helix seems to align perpendicular to the surface on negatively charged vesicles, as indicated by the small negative peak at ~215 nm, probably shifted from ~210 nm, between two large positive signals (Figure 3B). The LD signal intensity in the 210–250 nm region followed the order LL-37, FK-16 > Tempo-La > Lasio III > Macro1, which could be correlated with the helix content detected by CD above. Furthermore, the reduced LD signal of all absorption bands in this region in the presence of neutral lipid bilayers might indicate a decreased overall alignment due to the less disruptive effects of the peptides on the lipid order [79]. The $n\to \pi^{*}$ transition in an α-helical conformation is perpendicular to the helix axis and appears at ~210–230 nm in the LD spectrum [84] (Figure 3C). This infers that the sign of the LD peaks at ~210 nm and at ~220–230 nm should be opposite since the corresponding transitions are orthogonal. In line with this, the peptides with a positive LD at ~210 nm exhibited negative peaks at 220–230 nm (Figure 3A and Figure S2A–C), further supporting a helical state, aligned parallel to the membrane plane, except for the peptide Tempo-La as mentioned before (Figure 3B). In the case of Lasio III and Tempo-La, however, the band at 220–230 nm cannot be assigned solely to the amide $n\to \pi^{*}$ transition since the aromatic residues, Trp in Lasio III and Tyr in Tempo-La, also absorb in this region. Thus, these peptides were analysed in more detail, also utilizing other methods reporting sensitively on these side chains.

In the case of Lasio III, the LD peaks corresponding to the transition moments of the indole ring moiety (Figure 3A, inset) can be analysed. The intense negative LD peak at 228 nm suggests that the tryptophan $B_{b}$ transition moment is oriented essentially perpendicular to the membrane surface (Figure 3A). Conversely, both the broad and unstructured $L_{a}$ band at ~250–270 nm and the vibrational structured $L_{b}$ band at ~290 nm display positive LD signals (Figure 3A), which are indicative of an orientation parallel to the membrane surface [81,85]. All of these point to a tryptophan interplay at the membrane interface where the most hydrophobic part of the side-chain is oriented towards the membrane interior, while the electronegative nitrogen is towards the polar lipid head-groups [79]. This was further supported by intrinsic peptide fluorescence where, upon membrane binding, the Trp emission maximum blue-shifted from 348 nm to 325 nm (Figure 3D), which is characteristic for a Trp residing in a rather apolar microenvironment [86,87]. Thereby, the effect presumably includes the incorporation of the side chain into the membrane, which was more pronounced with the negatively charged liposomes. The combined LD and fluorescence data suggest that while the helical peptide lies parallel to the surface of the membrane, the Trp residue points away from the helix axis towards the hydrophobic membrane interior. In contrast, for the PC-bound peptide, the rather wide fluorescence emission peak, with only a slightly blue-shifted maximum compared to the free peptide, might indicate tryptophan populations in solvent-exposed environments [88].

Tempo-La contains a single Tyr residue where the side-chain phenol group possesses two orthogonal in-plane transition moments (Figure 3B, inset). The absorbance peaks at ~230 nm ($L_{a}$ band) and at ~280 nm ($L_{b}$ band) correspond to the transition moments polarized along the long and the short axis of the phenol ring, respectively. The positive LD signal was detected at 228 nm, which also corresponds to the $L_{a}$ band, indicating that the chromophore might lie parallel to the membrane surface. Therefore, special focus should be put in the near UV region where only the aromatic residues contribute; a positive LD signal was clearly detected for the $L_{b}$ band at 276 nm (Figure 3B), which is indicative of a chromophore lying parallel to the membrane surface [78,80]. According to signal intensities, the phenol ring has the most defined positioning with PC/PS. Based on LD spectral features considering helix orientations detailed above, it is possible to hypothesize a unique binding mode for Tempo-La where the helical part is rather parallel with the membrane normal with PC/PG, while rather parallel with the membrane plane with PC, and can bobble between these two orientations with PC/PS. This idea is supported by simulation results (see below, Figure 6B,C) showing an “antenna-like” binding mode upon binding to PC/PS where the C-terminal helical part can freely move around the N-terminal anchoring segment. In addition, intrinsic peptide fluorescence data utilizing Tyr fluorescence (Figure 3E) provided further details. In the presence of PC/PG and PC/PS vesicles, an increase in emission intensity was detected compared to the non-bound peptide. This implies that the fluorescence of the peptide is quenched in the absence of lipids, presumably due to the proximity of Tyr residues in a self-assembled state. Indeed, we could detect peptide associates by DLS (Figure S3A). However, interaction with lipids forces the peptides to dissociate and bind separately to the vesicles. Interaction of the Tyr group with the vesicles was further indicated by IR data based on shifts of the Tyr ν(CC) ring vibration band from 1516 cm^−1^ of the free peptide to 1518 cm^−1^ observed in the presence of the liposomes (Figure 3F).

### 2.4. Effect of the Peptides on the Size and Morphology of Model Vesicles

**Figure 4:**
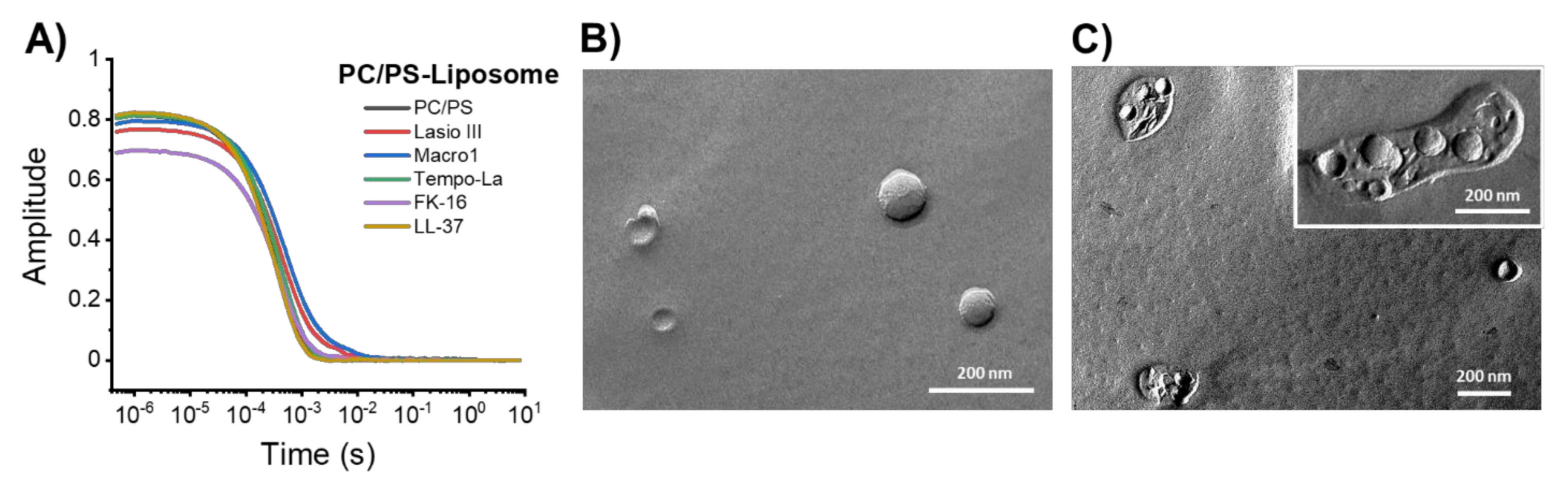
Size and morphology variations of the vesicles induced by the ACPs. (**A**) Correlation function by DLS for the studied ACPs in the presence of the lipid membrane PC/PS; peptide and lipid concentrations are 40 and 635 µM for all the peptides, except for LL-37, which is 20 and 320 µM, respectively. Freeze fractured TEM images of (**B**) PC/PS-alone; lipid concentration 2540 µM and (**C**) LL-37-PC/PS mixture; peptide and lipid concentration are 80 and 2540 µM, respectively.

From the molecular level effects of the ACPs on the lipid bilayers above, we also examined macroscopic effects, thus testing changes of vesicles in size and morphology. DLS data indicated that peptide binding hardly affected liposome integrity to a detectable level. No significant changes were detected for PC and PC/PG liposomes (Figure S3B,C), and only Lasio III and Macro1 induced shifts of the correlation function with PC/PS liposomes (Figure 4A). The latter is indicative of an emerging vesicle subpopulation of ~150 nm compared to the 100 nm-sized intact vesicles, where the liposomes were presumably bridged by the peptides. Although no vesicle aggregation was detected for LL-37 using DLS, FF-TEM images clearly demonstrated such an effect with PC/PS liposomes. The pure lipid system was characterized by isolated, spherical-shaped, ~100 nm-sized, unilamellar vesicles (Figure 4B), while the interaction with LL-37 promoted the contacts between the lipid vesicles, leading to a “shell-packed” morphology (Figure 4C) with a group of nearby vesicles enclosed in a shell-like envelope. Previous investigations on the cationic peptide DHVAR4 with anionic membranes showed similar behaviour, where small groups of closely located vesicles were embedded into larger shell-like structures [89].

### 2.5. Molecular Dynamics Simulations on the Peptide-Lipid Interactions

To further address peptide-lipid interactions on the molecular level, molecular dynamics (MD) simulations were also carried out. Peptides were placed approximately three nanometres away from the surface of the model membrane (PC, PC/PG, or PC/PS) and simulated for 500 nanoseconds. Based on the CD results above, the initial secondary structure was chosen to be helical.

**Figure 5:**
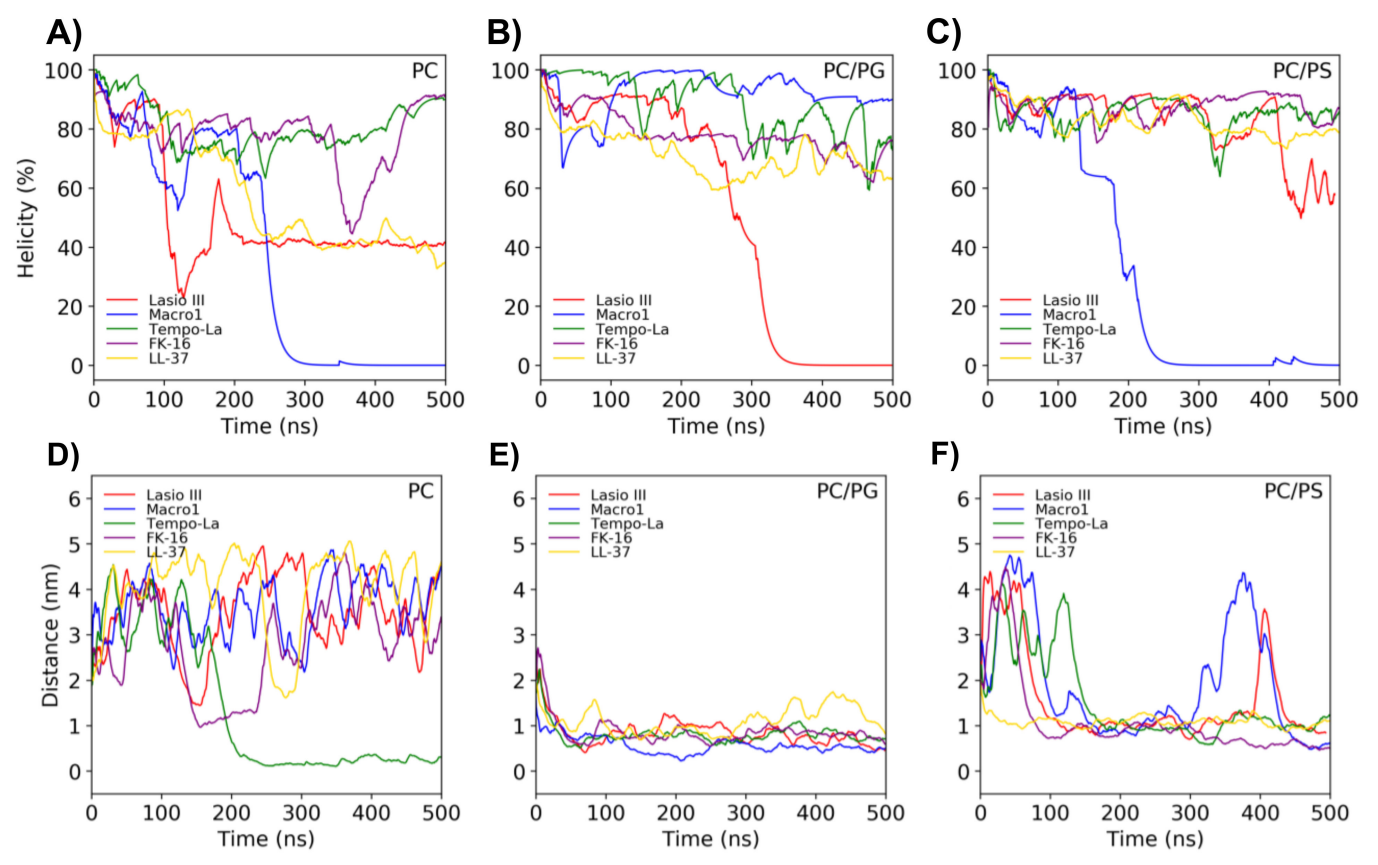
Peptide helicity changes in the presence of model membranes and distance of the ACPs from the bilayer during the MD simulations. The helicity of the peptides in the presence of the model membranes (**A**) PC, (**B**) PC/PG and (**C**) PC/PS is defined as the number of the residues with α-helical structure divided by the total number of the residues minus the two terminal residues (for further information see the methods section). The distance of the peptides from the surface of the different model membranes (**D**) PC, (**E**) PC/PG and (**F**) PC/PS as a function of the simulation time.

First, the helicity was monitored over the simulation time (Figure 5A–C and Figure S4), which provides an indirect way to study bilayer-induced conformational changes without tedious rare event sampling methods. The helix content of the peptides showed remarkable differences with respect to the applied model membrane. Lasio III exhibited the most peculiar behaviour. Its secondary structure remained mostly helical in the presence of the PC/PS membrane, and it still retained some helicity with the PC bilayer; in contrast, helicity was lost with the PC/PG bilayer (Figure 5B). It should also be noted that Lasio III was the only peptide for which the presence of the PC/PG bilayer hampered peptide helicity instead of promoting it. Although this is somewhat contradictory to CD results, where some helical conversion was observed with the PC/PG liposome, however, the helix content was the lowest for this peptide. On the other hand, Macro1 remained helical only in the presence of the PC/PG bilayer and lost helicity with the PC and PC/PS bilayer (Figure 5A,C). Tempo-La and FK-16 retained most of their helicity for the entire course of the simulation, irrespectively of the model membrane (Figure 5A–C). Finally, LL-37 retained some helix content with the PC liposome, but higher helicity is observed with PC/PG and PC/PS membranes (Figure 5B,C).

Moreover, peptide distance from the bilayer surface was also calculated as a function of the simulation time (Figure 5D–F and Figure S5). Interestingly, none of the peptides were found close to the PC bilayer, except for Tempo-La at >200 ns (Figure 5D), which is in line with the LD results suggesting that Tempo-La resides parallel to the membrane surface of PC. On the other hand, the most straightforward interpretation comes with the PC/PG membrane because all the studied peptides bound almost immediately and irreversibly onto the bilayer surface as indicated by the small peptide-surface distances (Figure 5E). Likewise, this behaviour remained characteristic for the PC/PS bilayer as well; however, peptide binding was less immediate and irreversible, especially for Lasio III and Macro1 (Figure 5F). 

**Figure 6:**
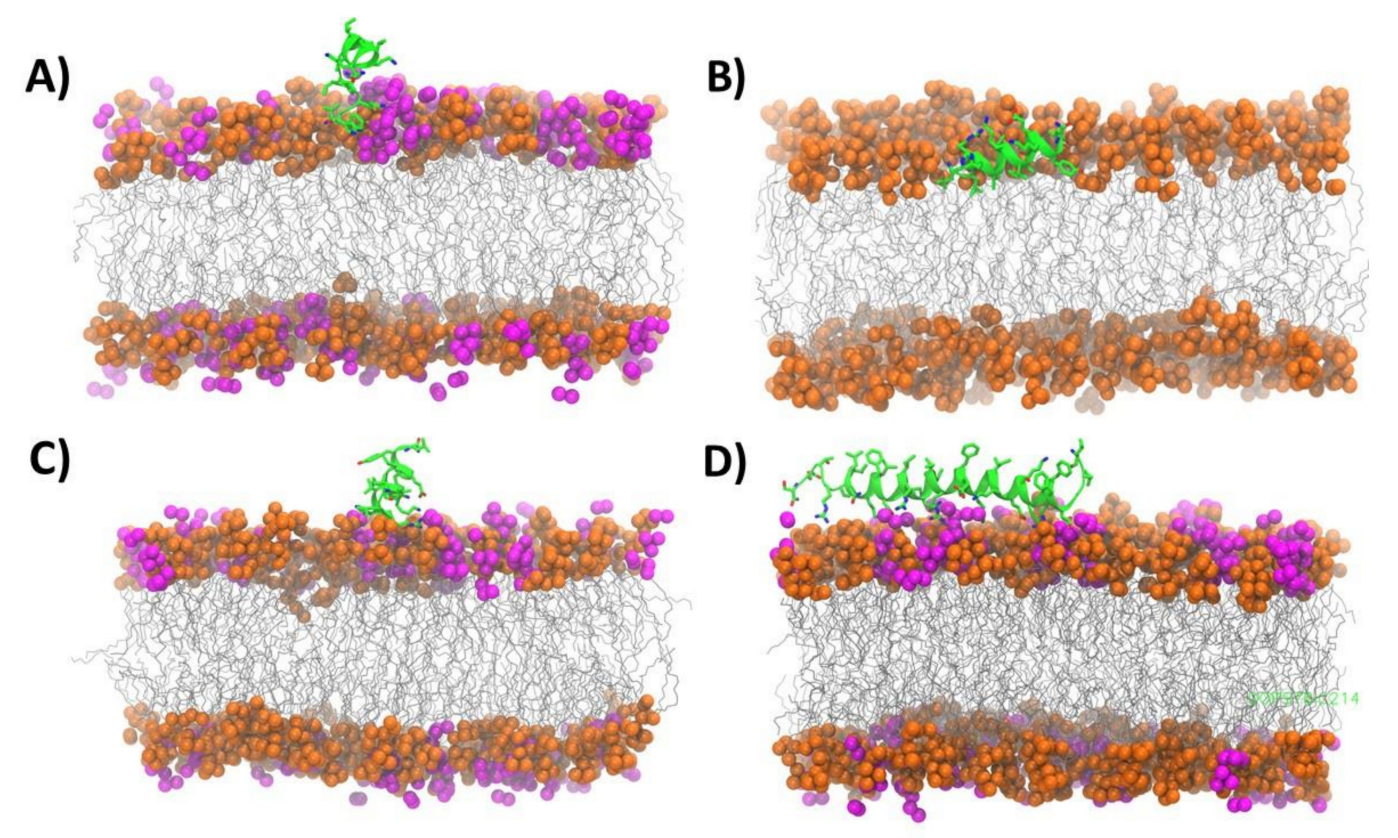
Membrane orientation of selected ACPs in the MD simulations. (**A**) Lasio III in the presence of PC/PS bilayer. (**B**,**C**) Tempo-La in the presence of PC and PC/PS bilayer, respectively. (**D**) LL-37 in the presence of PC/PS bilayer. The backbone of the peptides is shown as green cartoon. The lipid head groups are represented by orange (PC) and magenta (PS) spheres, while the lipid alkyl chains are grey lines. The H-atoms are not shown for better clarity. Representative snapshots were taken at the end of the simulations (at 500 ns).

The combined results from MD simulations revealed several details regarding the peculiar binding mode of the studied ACPs. A unique behaviour was detected for Lasio III that lost its helicity upon binding to the PC/PG surface, oppositely to all the other peptides studied here, which indicates a potentially different mechanism for this peptide, as it was able to bind to the other negatively charged liposome PC/PS (Figure 6A). It was also observed that, as indicated by LD, the Trp of Lasio III is oriented towards the inside of the bilayer (Figure 6A). Furthermore, Tempo-La was able to bind even to the zwitterionic PC membrane (Figure 6B) and inserted the deepest among all the other peptides, which is potentially the reason for its higher helicity. Moreover, a unique orientation can be observed for this peptide with PC/PS; namely, it is attached to the membrane surface, but its helix is oriented perpendicular to the membrane surface (Figure 6C), as also suggested by LD data. In the case of the cathelicidin peptide and its fragment, the interaction of LL-37 fits the general picture obtained experimentally, as it selectively binds to negatively charged PC/PG and PC/PS (Figure 6D), where it shows pronounced helicity. FK-16 retained its helicity in the presence of all model membranes studied, even though it did not bind to the surface of the PC bilayer, which indicates its high affinity for a helical structure.

### 2.6. Interaction of ACPs in a More Complex Membrane System

To better understand the effect of the investigated ACPs on biomembranes, a more complex membrane system was also applied. Previous studies have suggested that red blood-cell derived extracellular vesicles (REVs) can be used as improved model systems to study membrane interactions, as they can be produced on a large scale, are non-toxic, and have a monodispersed size distribution making them suitable for biophysical experiments [90,91]. We have demonstrated that membrane-active peptides target not only the lipid bilayer of extracellular vesicles but also that their unique action can result in the removal of EV-associated protein corona members [92]. To improve our mechanistic understanding, LL-37, FK-16, and Tempo-La were selected, and their interactions with REVs were studied by increasing ACP concentration and monitoring with LD spectroscopy.

**Figure 7:**
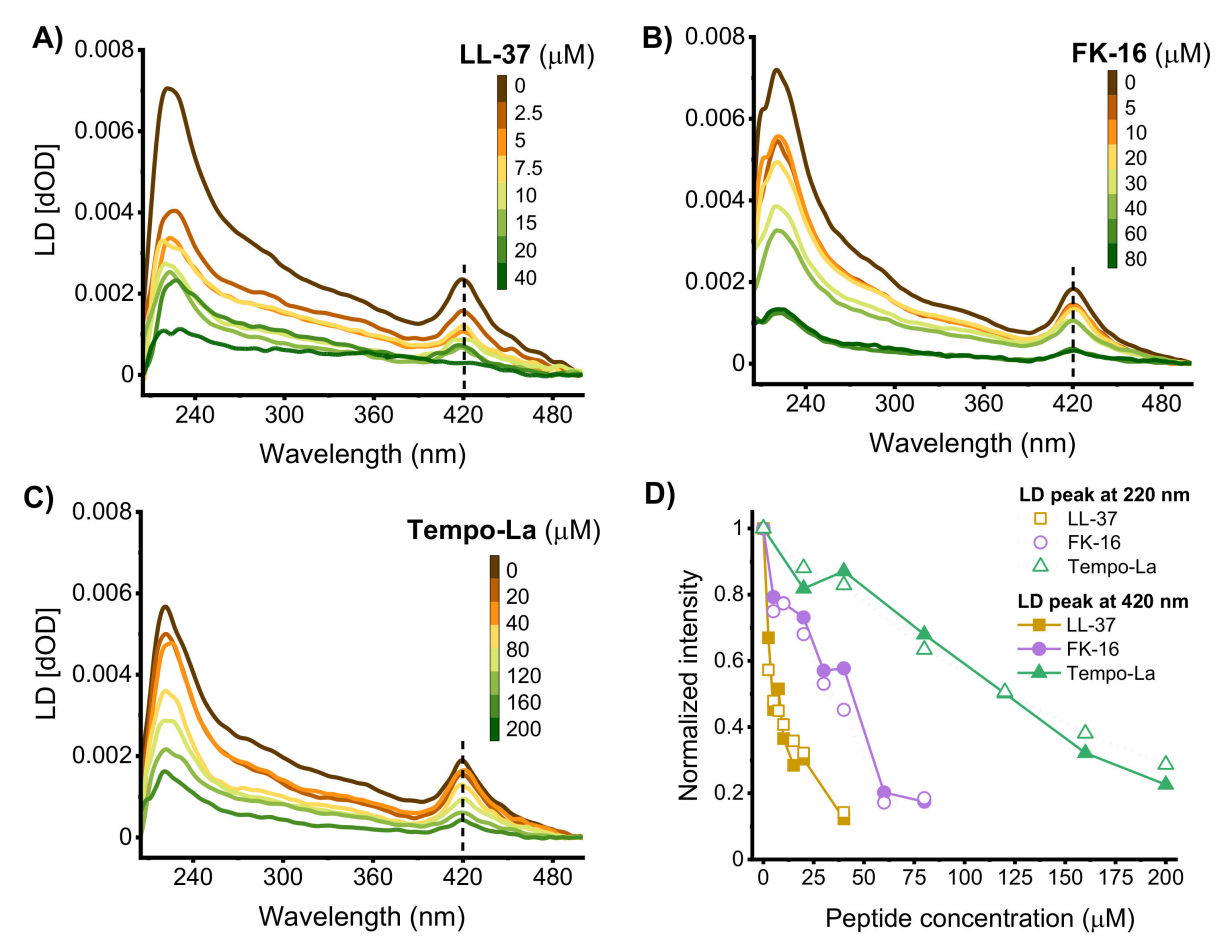
REV-ACP interactions studied by LD spectroscopy. The Soret band in the LD spectra of REVs is indicative on how ACPs interact with the surface of the vesicular bilayer. LD spectra of REVs upon addition of (**A**) LL-37, (**B**) FK-16, and (**C**) Tempo-La. (**D**) LD peak intensity at 420 nm (close symbols) and at 220 (open symbols) as a function of the ACPs. Data are normalized to values measured in the absence of peptides.

The LD spectrum of free REVs (Figure 7A–C) displays a major positive band at ~220 nm arising from a mixture of the $n\to \pi^{*}$ and $\pi \to \pi^{*}$ amide transition of REV proteins and a peak with a lower intensity at ~420 nm corresponding to the Soret band of heme proteins, which, in the case of REVs is haemoglobin [84,93]. Titration of REVs with ACPs resulted in a substantial decrease of both LD bands at 220 nm and 420 nm, showing the most abrupt change for the peptide LL-37 (Figure 7A). At a concentration of 5 μM, LL-37 has already reduced both main absorption bands by ~50% compared to the control REVs, and its further addition up to 40 μM led to complete loss of the LD signal (Figure 7D). A similar trend was observed for FK-16, though higher peptide concentrations were needed to reach the same intensity decrease (Figure 7B,D). In contrast, no significant change was observed for Tempo-La at 40 μM, and ~25% of the haemoglobin peak was still detected at 200 μM (Figure 7C,D). These results indicate that the ACPs applied here could affect REV associated proteins, even when the effective concentration seems to depend on the peculiar peptide.

Structural effects upon REV-ACP interactions were also followed by CD and IR spectroscopy. Very similar spectral changes were detected here (Figure S6 and related text in SI) as observed previously on melittin and CM15 [92], which is indicative of a common action exerted by this set of peptides. All these findings suggest that the novel function, e.g., the removal of REV-associated proteins revealed for melittin and its hybrid CM15, can be extended to several cationic membrane-active peptides. However, the ACPs applied here could affect REV associated proteins to a different extent, presumably depending on their type of interaction mechanism.

## 3. Discussion

In the activity of membrane-active peptides, selectivity towards a particular membrane composition is a key factor. We found that the five studied ACPs clearly differ in their lipid preference. Overall, the peptides showed preferred binding to PC/PG and PC/PS over pure PC liposomes. Particularly, moderate helical folding was observed for Lasio III, Macro1, and LL-37 in CD experiments. Reduced helicity was supported by MD simulations, in which these three peptides lose helicity by the end of the employed simulation time (Figure 5A). This behaviour is in line with the well-known fact that positively charged peptides often show lower affinity towards neutral, zwitterionic phospholipids [94], from which the outer leaflet of the mammalian cell membrane is exclusively composed [95,96]. In contrast, Tempo-La and FK-16 showed high helicity even in the presence of PC liposomes, as indicated by both CD and MD results (Figure 1F and Figure 5A). These suggest that for the latter two ACPs, electrostatic interactions with the polar groups of the lipids could be stronger. Among the negatively charged vesicles, Lasio III, Macro1 and Tempo-La preferred PC/PG liposomes while FK-16 preferred PC/PS liposomes. In contrast, LL-37 displayed a preference towards neither PG nor PS liposomes (Figure 1F). These findings show that their membrane interaction depends not only on simple electrostatics but the spatial distribution of the charged moieties within the lipid headgroup and along the peptide sequence can fine-tune their binding. As cancer cells are characterized by exposed PS, peptide preference for PS argues for good anticancer activity. Indeed, FK-16 is known for its excellent anticancer efficiency [35,36]. However, FK-16 showed comparable folding in the presence of PC and PC/PG liposomes, which might be attributed to its high helix propensity. The latter is supported by the helical wheel projection showing an amphipathic helix with perfect separation of the hydrophilic and hydrophobic residues (Scheme 1A). Thus, the high intrinsic helicity of the peptide promotes interaction and helical folding even with PC bilayers. For its parent peptide, LL-37, some hydrophobic residues insert in the hydrophilic side. Moreover, several negatively charged residues and helix breaker residues are present, which can lead to a loosening of the helical conformation and, in turn, to enhanced selectivity towards negatively charged membrane surfaces, as observed here. Out of the studied peptides, Lasio III showed the lowest helix content (Figure 1F) for all three model membranes used. The experimental observation is in full line with the qualitative results of the MD simulations, as this peptide was shown to lose helicity during the simulations (Figure 5A–C).

To better understand the relationship between sequence and mode of membrane association, it is also important to consider that the studied ACPs change their initial structure to optimize their interaction with membranes. This conformational change is proposed to increase the amphipathicity of the membrane-bound peptide [97]. The latter property is strongly related to their hydrophobicity and net charge, and this could affect peptide activity, target selectivity, and toxicity towards eukaryotic cells [9]. Based on the mean hydrophobicity (H) of their sequence and the mean hydrophobic moment $\left(\mu_{H}\right),\;$in their α-helical form [49,50,98], many of the studied ACPs have a strong tendency to reside on the surface of the membrane. FK-16 and Tempo-La are the two ACPs out of the five which have the highest helicity upon membrane association. Interestingly, based on the helical wheel model (Scheme 1A), and their $\mu_{H}$ and H values (Table 1), they are rather similar. However, current results demonstrate that these peptides are oriented preferentially perpendicular to each other on the membrane surface. A particular difference between the two peptides could be that for Tempo-La, the apolar residues are somewhat dominating (8 over 5), whereas, for FK-16, these are more evenly distributed (8 to 8). The MD simulations also show that for Tempo-La, its’ LLR N-terminal motif has a high affinity to the lipid headgroup region, with the Arg side chain having multiple hydrogen bonds formed when coordinating on the phosphate and carboxyl groups of the PG and PS lipids. Due to the longer size of the Arg side chain, the simultaneous surface presence of Leu-Leu and the Arg side chain can only occur in case the latter acts as a support on the side of the formed highly helical secondary structure, which thus positions the helix upwards initially. This, combined with the fact that the sequence is rather short (only 13 residues; FK-16 has 16), and has only three cationic residues (FK-16 has 5), will thus, result in reduced electrostatic attraction towards the membrane surface. Furthermore, although both Lasio III and Macro1 have high H and $\mu_{H}\;$ values favouring helix formation, their lower helical character could be linked to Gly residues in their relatively short sequences (15 and 13 aa for Lasio III and Macro1, respectively). Gly tends to disrupt helices because its high conformational flexibility makes the formation of the constrained α-helical structure entropically expensive. In the case of LL-37, some parts of the long sequence are expected to be very similar to FK-16, whereas other sequential regions, particularly with negatively charged residues, could have more distinct properties from common amphipathic ACPs and AMPs (Table 1). 

**Scheme 2:**
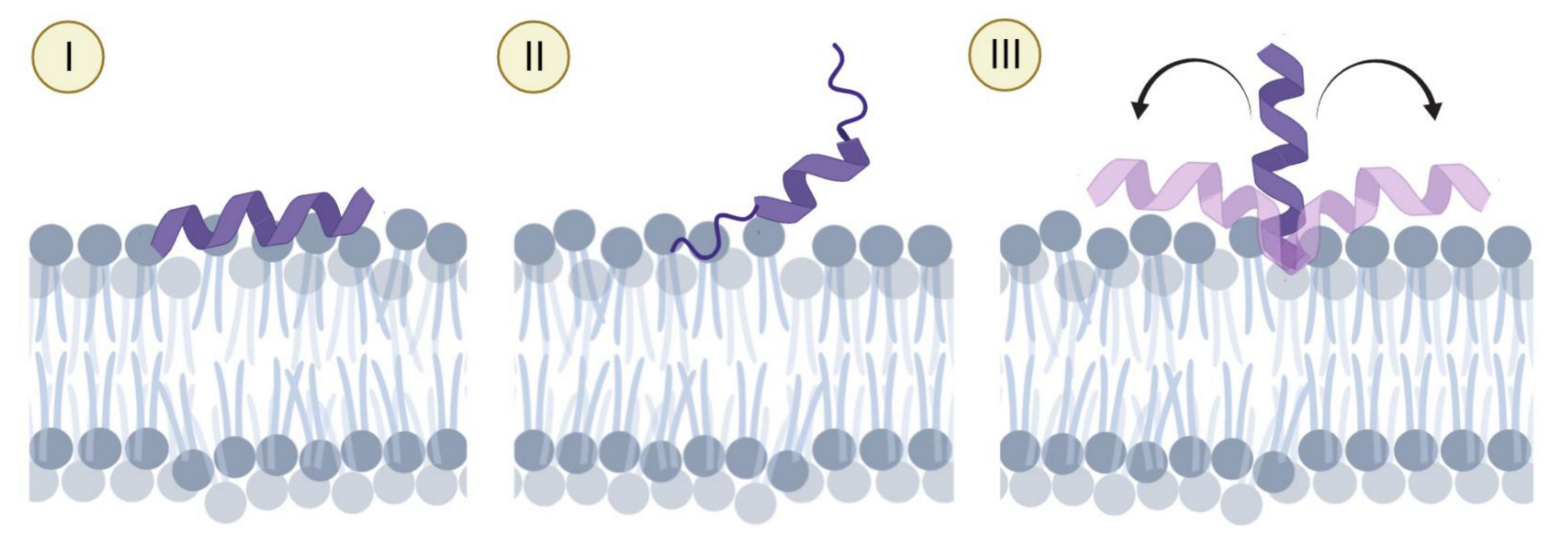
Proposed membrane-association mechanism for the peptides used in the study. (**I**) Highly helical, occupying a higher space on the surface in line with the general carpet model observed for FK-16 and LL-37. (**II**) Partially helical, still mostly lying on the surface observed for Lasio III and Macro1. (**III**) Highly helical, but preferentially perpendicular to the surface, occupying smaller space identified for Tempo-La.

Beyond the above observations and considerations on peptide helicity, it can already be seen for the case of Tempo-La that the combination of the employed experimental and computational techniques allowed further differentiation. In this respect, flow-LD spectroscopy and MD simulations prove to be particularly useful, as they provide structural information on the orientation of the helical ACPs relative to the membrane surface and fluorescence results and experiments with REV vesicles gave new insights on the surface affinity and association mode of the ACPs. Based on these, the five peptides investigated were categorized into three binding modes as follows (Scheme 2).

### 3.1. Highly Helical—Carpet Model 

This binding mode is proposed for FK-16, and to some extent, its longer precursor peptide LL-37, where the highly helical ACPs associate to the membrane surface via interacting mainly with the headgroup region of the lipids (Scheme 2). The helical parts of these peptides seem to be oriented preferentially parallel to the membrane surface, as indicated by LD (Figure S2B,C) as well as by the MD simulations. The latter method confirmed both high helicity and close attachment to the membrane surface (Figure 5). IR analysis also showed that they perturb the phosphate and choline groups of the lipids (Figure 2), suggesting their binding to the surface of the bilayer. Distinctive perturbations were also found for the lipid ester and acyl chain vibrations, especially for LL-37 with PC/PS liposomes, showing a strong membrane interaction affecting deeper regions of the bilayer perturbing lipid order and packing as well. The results with REVs suggested that they could efficiently remove the surface-associated protein corona in a concentration-dependent manner, which promotes that their helices covering the vesicle occupy a relatively large space on the EV surface [92] (Figure 7D). In this case, the longer LL-37 was proven to be more effective. Thus, this highly helical conformation, with the helix being parallel to the membrane surface, represents a binding mode that is compatible with the carpet model described for AMPs [15,99], in which the peptides accumulate on the surface of the target membrane.

### 3.2. Partially Helical, Surface Binding 

The second proposed binding mode was found for Lasio III and Macro1, which is similar to the first one to some extent; however, here, only a partial folding of the peptide is achieved upon interaction with the membrane (Scheme 2). This is clearly supported by CD (Figure 1A,B) and by their low-intensity LD spectra (Figure 3A and Figure S2A). In line with experiments, MD simulations also qualitatively demonstrate the significant decrease in helicity during the time course of the simulation for all lipid compositions studied (Figure 5A–C and Figure 6A). This strongly suggests that despite markedly increased helicity in the presence of negatively charged liposomes, these peptides have a lower inherent propensity to fold into a regular helical structure compared to the peptides of the first binding mode. However, IR showed that the perturbed phosphate group vibrations were particularly noteworthy, as was the impact of Lasio III on the polar moiety of all the examined lipid systems. The latter is in line with MD simulations on the PC/PS bilayer showing that the C-terminal helical part of Lasio III is out of the membrane while a disordered N-terminal segment bearing the Trp anchors the peptide (Figure 6A). The latter is in close agreement with fluorescence and LD results suggesting a Trp side chain buried in the lipid bilayer (Figure 3A,D). The former also promotes that both the helical and the disordered regions can have a role in interacting with the membrane. In this case, the simultaneous presence of different secondary structures could open alternative ways compared to the fully helical structures, where further steps, such as oligomerization by interactions between, i.e., the disordered segments, followed by membrane insertions, could be easier foreseen. Note that longer sequences, such as the bee venom melittin [79,100], or even LL-37 [31,37,61], will have some of their parts unfolded to some extent, and thus, belong more to mode II than I, depending on lipid composition and other environmental conditions. This may also provide more adaptability for longer sequences into various conditions, allowing potentially more widespread and diverse functionality. 

### 3.3. High Helicity, Non-Inserted, Perpendicular to Surface 

The third binding mode suggested here is for Tempo-La, which is significantly different from the first two. This ACP also adopts a helical conformation in the membrane-associated form according to CD and IR spectroscopy; however, as already discussed above, LD clearly demonstrates that the helix is perpendicular to the membrane surface (Figure 3B). Further, IR analysis of lipid perturbations (Figure 2), and MD simulations (Figure 6B,C), jointly suggest that this ACP is not inserted into the bilayer but is rather out of the membrane while attached to the lipids via its N-terminal part (Scheme 2). Based on simulations, in this binding mode, the helix has remarkable rotational flexibility, which allows the peptide to swing around in an antenna-like fashion. This distinct orientation can be best observed experimentally for PC/PS liposomes (Figure 3B), where the obtained LD signal is in line with the above binding mode, suggesting a preferentially perpendicular helix relative to the membrane surface. Based on MD, the ACP is attached to the surface by its LLR region, where the two N-terminal leucines are inserted more deeply into the headgroup region with connections to the hydrophobic chains, while the arginine side chain coordinates on the polar groups of the negatively charged lipids. Indeed, IR analysis revealed a slightly perturbed lipid phosphate region. Further on, intrinsic peptide fluorescence results (Figure 3E) indicate that Tempo-La is bound in a monomeric form to negatively charged membranes, as the quenching decreases in these cases with respect to the self-assembled non-bound peptide. This is further supported by measurements on REVs, as Tempo-La seems to be rather ineffective in removing the surface proteins compared to LL-37 and FK-16, indicating that it most likely occupies much less surface space (Figure 7D). In summary, these results all suggest that Tempo-La is bound to the negatively charged bilayer surfaces in an uncommon way, in a monomeric, helical form, where the helix is in a flexible standing position that does not cover much space on the surface. 

The observed categories clearly promote our understanding of different aspects of ACP membrane binding, which we hope provides further details on the structure-function relationships for ACPs. It should also be noted though, that these categories marked here can be clearly seen and identified for the shorter, 12–20 residue sequences; nevertheless, for longer peptides, such as LL-37, it is likely that there is more than one sequential region where binding modes can alter within the same peptide. MD simulations, in fact, show that LL-37 has parts partially unfolded while membrane-bound, indicating that some regions, i.e., that of FK-16, adopt a highly helical binding mode, where other segments correspond more to the partially helical binding mode. Indeed, the solution NMR structure of LL-37 in a membrane-mimicking environment showed disordered segments at both termini and a kink at Lys12, preceding Gly14, separating a short and a long helix along the sequence [101]. In solid-state NMR experiments on lipid bilayers, including PG, high peptide helicity comprising residues 11–32 was supported, and a membrane disruption model that evolved from initial carpeting of the membrane surface was suggested [61].

Furthermore, our results on peptides with binding mode II are also in line with reported data using simple model systems mimicking a bacterial membrane composition. A membrane association mode resembling the one proposed here was suggested for Lasio III, including surface association and an anchoring N-terminal part using PC and PC/PG model vesicle systems [102], and helix formation only for the C-terminal part and inserted Trp of the N-terminal segment in micellar membrane-mimicking environment [1]. In an NMR study [21], the peptide was shown to adopt a curved helical conformation in a helix-promoting aqueous solution, attributed to the flexible part around the central Gly residue, which was suggested to play roles in its selectivity. Likewise, a partially helical state with a C-terminal ordered segment was indicated to some extent for Macro1 bound to SDS micelles [26]. For Tempo-La, while the current binding mode was identified on model vesicles, it is a likely scenario that such a stage will be an intermediate state that would progress in the presence of biological membranes, such as those of cancer cells, into a final membrane inserted and/or oligomeric phase, which is directly responsible for the anticancer activity. This is supported by the fact that pore formation was observed for Tempo-La on bacteria [28], which mechanism might be facilitated via a parallel positioning of the peptides relative to the lipids as revealed here for this peptide with negatively charged liposomes. Alternatively, this binding mode resembles the snorkelling or shifted helix model described for membrane-spanning proteins where the cationic but also highly hydrophobic Arg/Lys side chains contribute to positioning a partially inserted helix near the membrane surface [103,104,105,106]. Note, however, that for the latter examples, the effect of other sequential regions is unknown; thus, this comparison remains speculative at this point. Related, the experimental data may allow the co-existence of two peptide populations with parallel and perpendicular orientation with respect to the bilayer, respectively. However, this scenario is less likely based on computational results. Nevertheless, this interesting phenomenon for Tempo-La is worth investigating in detail, but at this point is beyond our current focus.

In this study, we focus on model vesicles composed of a few phospholipids to characterize ACP-membrane interactions. Biomembranes contain a tremendous variety of lipids with very diverse physicochemical properties determining membrane properties, e.g., curvature, surface charge, and fluidity, hard to mimic by simplistic models. Nevertheless, the binding modes described here can readily explain ACP actions observed on REVs, a more complex model system representing physiological-like lipid composition, with extra protein content. It is expected that once EVs from various cancer cells can be produced in quantities suitable for the above structural studies, we will be able to see further insight into mechanistic details of membrane-active compounds and, in particular, identify which types of cell membranes have the highest affinity to bind certain ACPs, aiding the identification of their optimal application areas. Finally, not only the membrane model but also the assay conditions should be considered in studies on peptide-membrane interaction, as highlighted recently [20,107,108,109,110]. In summary, with a critical view, the results presented here may provide a good assumption towards the in vivo action of the ACPs studied.

## 4. Materials and Methods

### 4.1. Peptide Solutions 

Lasioglossin LL-III (MW = 1765.6), Macropin 1 (MW = 1416.8), Temporin-La (MW = 1622.6), FK-16 (MW = 2044.5), and LL-37 (MW = 4492.8) were synthesized by NovoPro Bioscience Inc. (Shanghai, China). Peptide purity corresponded to ≥ 95%. For biophysical measurements, the lyophilized powder was dissolved in high purity water at 1 mM, aliquoted and stored frozen at −18 °C.

### 4.2. Lipid Solutions

High purity synthetic 1,2-dioleoyl-sn-glycero-3-phosphocholine (DOPC) and 1,2-dioleoyl-sn-glycero-3-[phospho-rac-(1-glycerol)], sodium salt (DOPG) was purchased from NOF (Tokyo, Japan) and 1,2-dioleoyl-sn-glycero-3-phospho-L-serine, sodium salt (DOPS) was purchased from Avanti Polar Lipids Inc (Sigma-Aldrich, Budapest, Hungary). Liposomes were prepared by using the lipid thin film hydration technique. Lipids were dissolved in chloroform (LabScan, Budapest, Hungary) containing 50% vol methanol (Reanal, Budapest, Hungary), which was then evaporated using a rotary evaporator. The resulting lipid film was kept in a vacuum for at least 8 h to remove residual traces of solvent. The dried lipid film was hydrated with the assay buffer. After repeated heating (37 °C) and cooling (−196 °C) steps (at least 10 times), the solutions were extruded through polycarbonate filters with 100 nm pore size (at least 11 times) using a LIPEX extruder (Northern Lipids Inc., Burnaby, BC, Canada). Final lipid concentration was 13 mM. For mimicking mammalian, bacterial, and cancer cell membranes, pure DOPC, DOPC/DOPG (80/20 n/n%), and DOPC/DOPS (80/20 n/n%) referred to as PC, PC/PG, and PC/PS, respectively, were used thoroughly in the study.

### 4.3. Assay Conditions

The assay buffer used thoroughly in the study to mimic physiological conditions was isotonic phosphate-buffered saline (PBS, 10 mM phosphate, 137 mM NaCl, 3 mM KCl, pH 7.4), purchased from Sigma-Aldrich (Budapest, Hungary). For measuring CD spectra, a buffer avoiding chloride ions (10 mM Na-phosphate, 100 mM Na_2_SO_4_, pH 7.0, CD buffer) was used frequently, which allowed spectra collection down to 190 nm. In the case of LL-37, CD spectra were recorded in Tris-HCl buffer (10 mM, pH 7.4). Furthermore, for collecting LD spectra, PBS containing 50% wt sucrose was employed as sucrose has the advantage of reducing light scattering of liposomes by matching their refractive index [77].

### 4.4. Red Blood-Cell Derived Extracellular Vesicles (REVs) Isolation

Blood was collected from healthy adult volunteers (~15 mL) in K_3_EDTA containing tubes (Greiner Bio-One, Kremsmünster, Austria). The use of human blood samples was approved by the Scientific and Research Ethics Committee of the Hungarian Medical Research Council (ETT TUKEB 6449-2/2015), and during all procedures, we followed the guidelines and regulations of the declaration of Helsinki. Red blood cells (RBCs) were pelleted by centrifugation at 2500× *g* for 10 min at 4 °C and washed three times with a physiological salt solution to achieve complete removal of buffy coats. RBCs were diluted with an equal volume of PBS and kept for 7 days at 4 °C, allowing REVs production. After this period, RBCs and cellular debris were removed in two subsequent centrifugation steps, first at 2500× *g* for 15 min followed by 3000× g for 30 min at room temperature. The supernatant of the second centrifugation step containing REVs was further centrifuged at 16,000× *g* for 30 min at 4 °C. The final pellet was resuspended in 100 μl PBS and purified with size-exclusion chromatography (SEC) using a 3.5 mL gravity column filled with Sepharose CL-2B gel (GE Healthcare, Danderyd, Sweden). The REV sample was pipetted onto the column, followed by the addition of 900 µL PBS while the flow-through was discarded. The purified REVs were eluted with PBS and collected in 1 mL. The samples were stored at 4 °C and used within 72 h after isolation. We performed standardized biophysical characterizations for each REV preparation using the spectroscopic protein-to-lipid ratio determination based on IR bands of the lipid CH and protein amide bands [68], which shows that this ratio is highly reproducible between preparations.

### 4.5. Circular Dichroism (CD) Spectroscopy

CD spectra were collected using a JASCO J-1500 spectropolarimeter at room temperature. CD spectra for peptide-liposome samples were collected in a 0.1 cm path-length cylindrical quartz cuvette (Hellma, Plainview, NY, USA) in continuous scanning mode between 190 and 260 nm at a rate of 50 nm/min, with a data pitch of 0.5 nm, response time of 4 sec, 1 nm bandwidth, and 3 times accumulation. CD spectra for the peptide-REVs samples were recorded on a Jasco J-715 spectropolarimeter in a 0.1 cm path-length rectangular quartz cuvette (Hellma, Plainview, NY, USA), using a continuous scanning mode between 197 and 270 nm at a rate of 100 nm/min with a data pitch of 1 nm, response time of 2 sec, 2 nm bandwidth, and 2 times accumulation. All spectra were corrected by subtracting a matching blank. To estimate the secondary structure of the peptides, the software BeStSel (http://bestsel.elte.hu) [67] was used. 

### 4.6. Linear Dichroism (LD) Spectroscopy

LD is defined as the differential absorption, A, between orthogonal forms of plane-polarized light, where the polarization vector of the incident light beam is oriented parallel ($A_{ǁ}$) and perpendicular ($A_{⊥}$) to the orientation axis of the sample:

(1) $$LD=A_{ǁ}-\;A_{⊥}$$

*LD* is used with systems that are either intrinsically oriented or are oriented during the experiment [66,83]. The sign and amplitude of the *LD* signal at a particular transition depend on the direction along which the light passes through the oriented sample. In a macroscopically aligned system, chromophores will exhibit *LD* if their transition moments have a preferential orientation relative to the orientation axis of the system. The alignment of liposomes can be achieved by shear flow in a rotation Couette cell device, resulting in ellipsoidal vesicles which align in the flow. Peptides that bind to the lipid surface in a non-random way will hence also be aligned, and their transition moments will exhibit *LD* in the 190–300 nm spectral region [78,111,112]. Linear dichroism measurements were performed on a JASCO J-1500 spectropolarimeter equipped with a Couette flow cell system (CFC-573 Couette cell holder) with a total path length of 0.5 mm. Spectra for the peptide-liposome samples were recorded between 195 and 400 nm at a rate of 100 nm/min with a data pitch of 0.5 nm, response time of 1 s and 1 nm bandwidth. The *LD* spectra for the peptide-REVs samples were collected between 195 and 600 nm at a rate of 100 nm/min with a data pitch of 0.5 nm, response time of 1 s, and 1nm bandwidth. The peptide-liposome and peptide-REV samples were oriented under a shear gradient of 2270 s^−1^. Baselines at zero shear gradient were measured and subtracted from all spectra. 

### 4.7. Dynamic Light Scattering (DLS) 

Samples were measured at 20 °C using a W130i dynamic light scattering device (DLS, Avid Nano Ltd., High Wycombe, UK) with a diode laser (660 nm) and a photodiode detector. Eppendorf disposable cuvettes (50–2000 µL, UVette routine pack, Vienna, Austria, GmbH) with 1 cm path-length were used. Samples containing 20 or 40 µM peptide and 320 or 635 µM lipid were measured in a final volume of 80 µL in CD buffer, except for LL-37, for which Tris buffer was used. The time-dependent autocorrelation function was measured for 10 s, repeated 10 times, and the average distributions were reported. Data analysis yielding the mean hydrodynamic diameter (Dh) and polydispersity were performed with the iSize 3.0 software supplied with the device.

### 4.8. Attenuated Total Reflection-Fourier Transform Infrared (ATR-FTIR) Spectroscopy

FTIR spectroscopic measurements were conducted using a Varian 2000 FTIR Scimitar spectrometer (Varian Inc., Palo Alto, CA, USA) fitted with a liquid nitrogen-cooled mercury-cadmium-telluride (MCT) detector and with a ‘Golden Gate’ single reflection diamond ATR accessory (Specac Ltd., Orpington, UK). Onto the diamond ATR surface, 5 µL of the sample was mounted, and the spectrum was collected (2 cm^−1^ resolution and 64 scans) for a dry film after slow evaporation of the buffered solvent under ambient conditions. Each data acquisition was followed by ATR correction, and buffer subtraction and baseline corrections were performed. For peak identification, spectra were normalized by the area, and the second derivative was calculated. Spectra analysis was performed using the Origin software package (OriginLab, Northampton, MA, USA). 

### 4.9. Transmission Electron Microscopy Combined with Freeze Fracture (FF-TEM)

For direct visualization of the structure and morphology of the sample, FF-TEM images were obtained with a JEOL JEM-1400 transmission electron microscope (JEOL Ltd., Tokyo, Japan) operating at 120 kV. Images were captured routinely at magnifications of ×15,000, 30,000, and 60,000 and analyzed with SightX Viewer Software (EM-15300SXV Image Edit Software, JEOL Ltd., Tokyo, Japan). In detail, a droplet (approximately 2 µL) of the samples prepared in PBS (peptide and lipid concentrations were 80 and 2540 µM, respectively) were pipetted onto a golden sample holder and rapidly frozen in liquid freon −194 °C, then put into liquid nitrogen. The fracturing was performed in a Balzers freeze-fracture device at −100 °C (Balzers BAF 400D, Balzers AG, Liechtenstein). A replica was made from the fractured surface with vaporized carbon-platinum. The replica was washed with surfactant solution and distilled water, and it was transferred to a 200 mesh copper grid with a support film made of formvar for measuring with the TEM equipment.

### 4.10. Fluorescence Spectroscopy

Fluorescence spectra for Lasio III and Tempo-La were recorded with a Jasco FP-8500 spectrofluorometer at 25 °C in PBS using 5 and 5 nm excitation and emission slits, respectively. The tryptophan fluorophore of Lasio III was excited at 295 nm, and the emission was monitored from 305 to 400 nm, while the tyrosine fluorophore in Tempo-La was excited at 275 nm, and emission was monitored from 290 to 400 nm. Binding assays were carried out at 2 µM peptide and 100 uM lipid. Blank spectra recorded for liposome solutions in the absence of peptides were subtracted.

### 4.11. Molecular Dynamics Simulations

All simulations were carried out by the GROMACS2018.3 [113,114] software, applying the CHARMM36m [115] force field. The initial, fully α-helical structures of Lasio III, Macro1, Tempo-La, FK-16, and LL-37 were created by Avogadro 1.1.1 software [116]. Similarly to the liposome models used in the experimental measurements, 100 n/n% PC, 80/20 n/n% PC/PG and 80/20 n/n% PC/PS bilayers were generated using the CHARMM-GUI website [117,118], and the bilayers consisted of 128 × 128 lipids and 2 nm water layer on each side, respectively. The peptides were simulated with all the bilayers; hence, a sum of 15 simulations were performed. The following simulation protocol was applied in each case. First, each peptide was inserted into a simulation box with the exact same a, b unit cell vectors as the box of the applied bilayer, while the c unit cell vector was chosen to be 5 nm. The simulation box was then solvated by TIP3P water molecules, and the coordinates of the atoms in the box were translated to be on the top of the bilayer box. The third box with water molecules was also created to extend the water layer by 4 nm at the bottom layer. The three boxes were then merged, neutralized by Na^+^ ions, and an excess of 150 mM Na^+^ and Cl^-^ ions were added to mimic better physiological conditions.

Next, the systems were minimized and equilibrated in six steps. First, the systems were minimized for 5000 steps using the steepest descent algorithm. In the following two steps, the systems were heated up to 300 K temperature and simulated for 75 picoseconds using berendsen and then V-rescale thermostats [119,120] (τt = 1.0 ps). In the last four steps, the systems were also coupled to a semiisotropic berendsen barostat [119] with 1 bar pressure (τp = 5.0 ps, κp= 4.5 × 10^-5^ bar^-1^) and simulated for 650 picoseconds altogether. The applied position restraints on the peptide-heavy atoms and lipid phosphorous atoms were gradually switched off during the six steps of the minimization/equilibration process.

To ensure that the correct statistical NPT ensemble is sampled, the systems were coupled to a Nosé-Hoover thermostat [121] with 300 K temperature (τt = 1.0 ps) and a semiisotropic Parrinello-Rahman barostat [122] (τp = 5.0 ps, κp= 4.5 × 10^-5^ bar^-1^) with 1 bar pressure during the production runs. Each system was simulated for 500 nanoseconds applying a 2 femtosecond time step. The electrostatics were treated by the Particle mesh Ewald (PME) method [123], and the cutoff was chosen to be 1.2 nm for the short-range electrostatic and van der Waals interactions. The LINCS algorithm [124] was used to constrain the hydrogen bonds. Periodic boundary conditions were applied in all directions.

The helicity of the peptides was calculated by the dssp method and defined as the number of the residues with α-helical structure divided by the total number of the residues minus the two terminal residues, which is inherently considered to be a coil, due to the applied algorithm. The distance between the peptide and the surface of the bilayer was defined as the absolute value of the distance between the z coordinate of the centre of mass (COM) of the peptide atoms and the z coordinate of the COM of the phosphorous atoms of that particular leaflet which is closer to the peptide. The analyses were performed using GROMACS internal packages (do_dssp, distance), bash and python scripts, and the MDAnalysis [125] python package. The snapshots were created by the VMD 1.8.2 software [126]. Exponential averaging was used to smooth the curves.

## 5. Conclusions

In this study, we have investigated the membrane association mechanisms of five natural helical peptides with known anticancer activity. ACPs represent a promising alternative to conventional chemotherapy. Nevertheless, a better understanding of their mechanism of action on target cancer cell membranes is needed to enable improved design strategies on their selectivity, to realize their therapeutic potential. In this work, we show clear differences in their helicity and related selectivity towards model vesicles mimicking healthy and cancer cell membranes. Further on, we propose three different membrane-association mechanisms for them, differing in the helix content of the peptides and the orientation of the helices relative to the membrane surface. LL-37 and its potent anticancer fragment FK-16 shared a common binding mode resembling the carpet model of classical antimicrobial peptides. Interestingly, peptide components of hymenopteran venom, Lasio III and Macro1, were classified to the same category, i.e., to a partially disordered binding mode on the surface, which suggests that these two peptides from similar sources dedicated to the same function share features in their action. Further, a unique mechanism and an “antenna-like” membrane association were revealed for Tempo-La, not yet described for membrane-active peptides. These findings provide further details into binding modes of the selected helical ACPs; however, it is expected that the identified categories can be applied to many other ACPs and, in general, also to antimicrobial peptides. Finally, it should be noted that, in line with recent results, the use of EVs as a more complex membrane system, incorporating host cell lipid and protein compositions, seem beneficial in gaining additional biophysical information on these systems, which cannot be obtained by simplified liposomal models. 

## Data Availability

Not applicable.

## Supplementary Materials

The following are available online at https://www.mdpi.com/article/10.3390/ijms22168613/s1.
