# Supplementary material for: Membrane Association Modes of Natural Anticancer Peptides: Mechanistic Details on Helicity, Orientation, and Surface Coverage
Source: Int J Mol Sci. 2021 Aug 10;22(16):8613. doi: 10.3390/ijms22168613 (PMC8395313; doi:10.3390/ijms22168613)
Supplement: Supplementary file 1 [file ijms-22-08613-s001.zip › ijms-1303600-supplementary.pdf]

Supporting Information

Membrane Association Modes of Natural Anticancer Peptides: Mechanistic Details on Helicity, Orientation, and Surface Coverage

Mayra Quemé-Peña[1,2], Tünde Juhász[1], Gergely Kohut[1,2], Maria Ricci[1], Priyanka Singh[1,2], Imola Cs. Szigyártó[1], Zita I. Papp[3], Lívia Fülöp[3], Tamás Beke-Somfai[1*]*

[1]Institute of Materials and Environmental Chemistry, Research Centre for Natural Sciences, Biomolecular Self-Assembly Research Group, Budapest H-1117, Magyar tudósok körútja 2, Hungary.
[2]Hevesy György PhD School of Chemistry, ELTE Eötvös Loránd University, Budapest H-1117, Pázmány Péter sétány 1/A, Hungary.
[3]Department of Medical Chemistry, University of Szeged, Szeged H-6720, Dóm tér 8, Hungary.

Table of contents

Figure S1. Infrared analysis of peptide secondary structure. 3
Figure S2. Peptide orientation upon binding to lipid membrane. 4
Figure S3. Lipid membrane size variations by the ACPs monitored by DLS 5
Table S1. Mean hydrodynamic diameter (Dh) and polydispersity of REVs samples. 5
Figure S4. Helicity of the studied peptides as a function of the simulation time. 6
Figure S5. Distance of the peptides from the surface of the different bilayers. 6
Figure S6. REV-ACPs interactions studied by CD and FTIR spectroscopy. 7

Infrared Analysis of Peptide Secondary Structure

Peptide conformational changes upon interaction with model membranes were further assessed by IR spectroscopy. The two prominent amide absorptions bands corresponding to the vibrational modes of C=O stretching (amide I ~1651 cm^{-1}) and N-H bending (amide II ~1540 cm^{-1}) around the peptide bond [1-3], was analyzed. The peptides in buffer (Figure S1A) showed a similar spectral profile of the amide I band, consisting of a main peak centred at ~1655/1660 cm^{-1} and a shoulder at ~1680-1689 cm^{-1}, which indicate α-helical/disordered conformations [4-6]. Lasio III has the most prominent shoulder (at ~1680 cm^{-1}) and the main peak centred at 1660 cm^{-1}, indicating the prevalence of turns and disordered conformations. The other ACPs exhibited a main peak centred at ~1655 cm^{-1}, which become narrower in the case of LL-37 and FK-16, indicating a higher contribution from α-helical structures.

Upon addition of zwitterionic PC liposome (Figure S1B), the intensity of the shoulder/band component at ~1680 cm^{-1}/1689 cm^{-1} clearly increased for all the studied peptides, this can be ascribed to the increment of intermolecular interactions in the presence of lipid membranes. In the presence of anionic PC/PG liposome (Figure S1C) Tempo-La, FK-16, and LL-37 in addition to the main intensity at ~1654 cm^{-1} (denotative of α-helical structure), also showed a shift of the shoulder ~1684 cm^{-1}. This latter might indicate peculiar extended conformations characteristic of molecules aggregated at the lipid surface, in this case an arrangement of peptide-lipid surface bound [7,8]. In addition, LL-37 and FK-16 exhibited two small contributions at 1616 and 1633 cm^{-1} that can be attributed to intermolecular interactions and aggregation [4,9]. These latter spectral features possibly suggest preferred interaction with PC/PG.

As observed with PC vesicles, also with PC/PS (Figure S1D) the main peak of Lasio III and Macro1 (~1657 cm^{-1}) indicated somewhat higher contribution from disordered structures compared to the other peptides. In accordance with CD spectroscopy results (Figure 1F), Macro1 and Lasio III showed lower helicity in favour of disordered conformations, as indicated by the amide I peak centered at ~1658 cm^{-1} in all the studied lipid systems. Moreover, it should be noted that Lasio III exhibited an overall distinct profile compared to the other peptides, suggesting a peculiar mode of interaction with lipid membranes. Thereby, the infrared analysis of the amide I region revealed that the tested lipid systems affected the peptide structure with noticeable differences, which might be ascribed to distinct mechanisms of interaction.

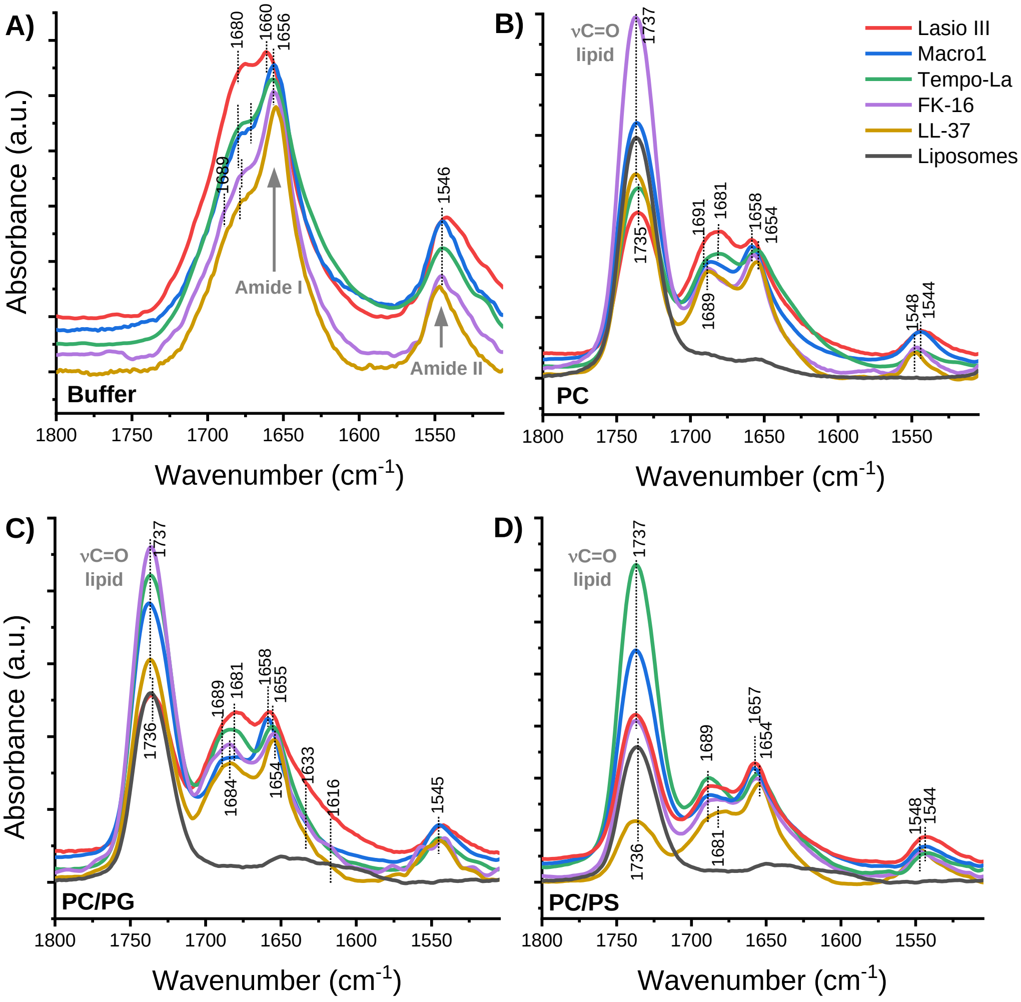

Figure S1. Infrared analysis of peptide secondary structure. Representative infrared spectra in the amide I and II region and the carbonyl C=O stretching vibration of the investigated peptides in (**A**) buffer and (**B**-**D**) in different model membranes. Spectra are normalized by the intensity of the amide I (~1655 cm^{-1}). Peak position and components of the amide I were identified by second derivative analysis. Peptide and lipid concentrations were 80 μM and 1.27 mM respectively, for all the peptides except for LL-37 which was 40 μM and 0.635 mM respectively, resulting in the same peptide-to-lipid ratio as for the other peptides.

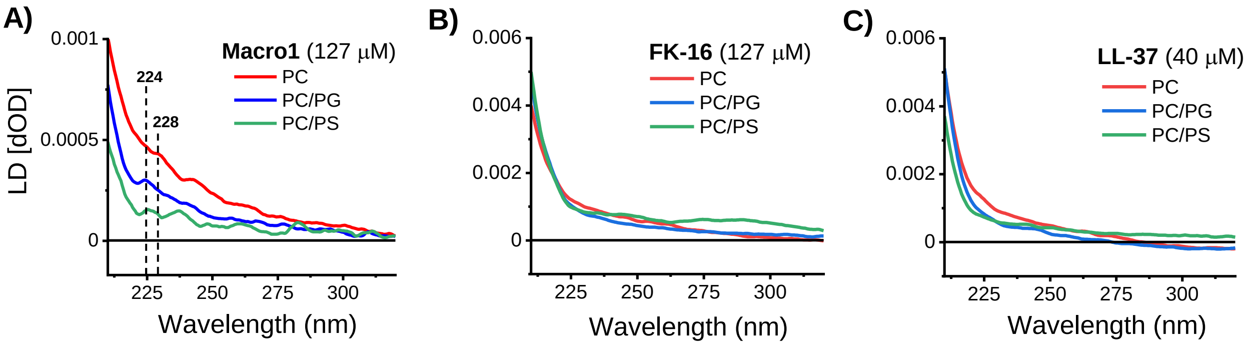

Figure S2. Peptide orientation upon binding to lipid membrane. LD spectra of selected ACPs (**A**) Macro 1, (**B**) FK-16 and (**C**) LL-37 in the presence of the model membranes PC, PC/PG and PC/PS. Spectra were collected at 1.27 mM lipid concentration.

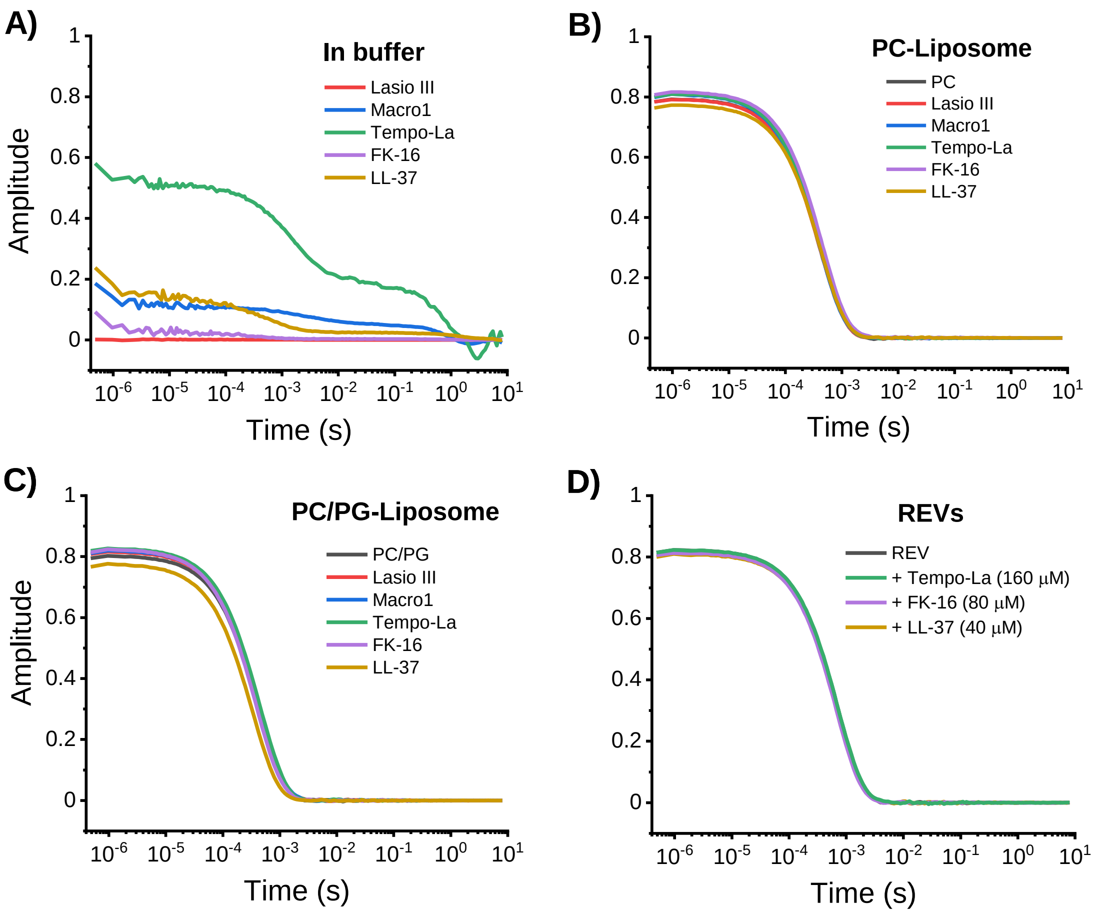

Figure S3. Lipid membrane size variations by the ACPs monitored by DLS. Correlation functions are shown for the studied ACPs in (**A**) buffer solution, (**B**) PC, (**C**) PC/PG and (**D**) REVs. Peptide and lipid concentrations were 40 and 635 µM respectively, for all the peptides except for LL-37 which was 20 and lipid was 320 µM, respectively. In the case of REVs the concentration of the peptides is indicated in the graph.

Table S1. Mean hydrodynamic diameter (Dh) and polydispersity of REVs samples. The concentrations used are the ones that induce ~90% in LL-37 and ~70% in FK-16, and Tempo-La reduction of the LD Soret band intensity.

Sample	D_h (nm)	Polydispersity (%)
**REV**	201 (± 35)	17
**REV + LL-37 (40µM)**	201 (± 41)	20
**REV + FK-16 (80µM)**	184 (± 18)	10
**REV + Tempo-La (160µM)**	204 (± 40)	20

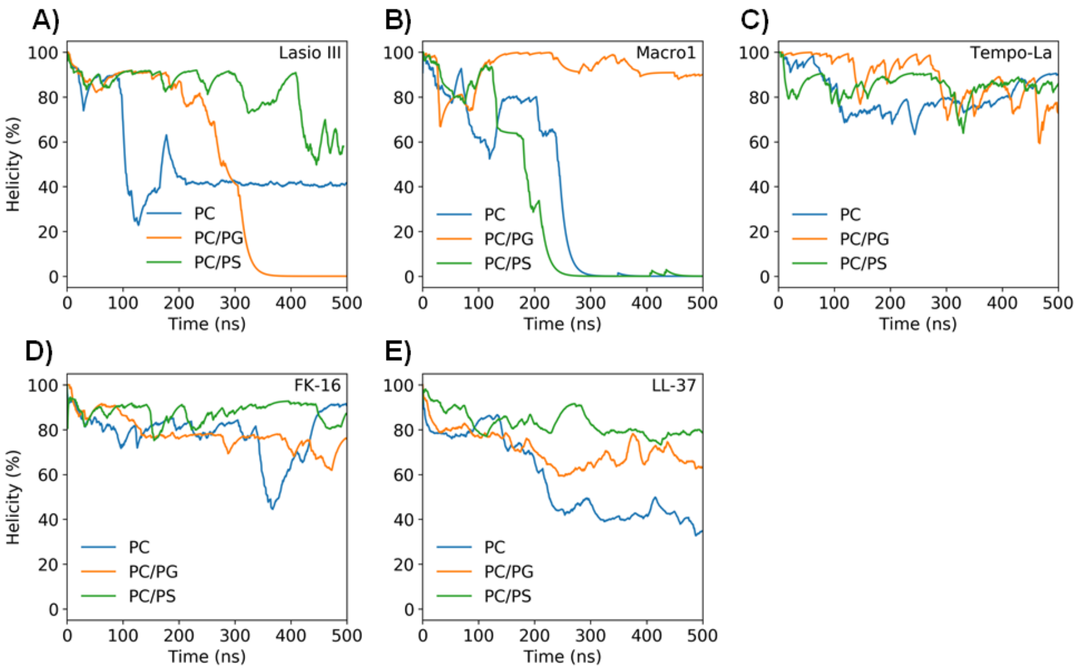

Figure S4. Helicity of the studied peptides as a function of the simulation time. The helicity of the peptides is defined as the number of the residues with α-helical structure divided by the total number of the residues minus the two terminal residues and has been grouped by each anticancer peptide used. (For further information see the methods section).

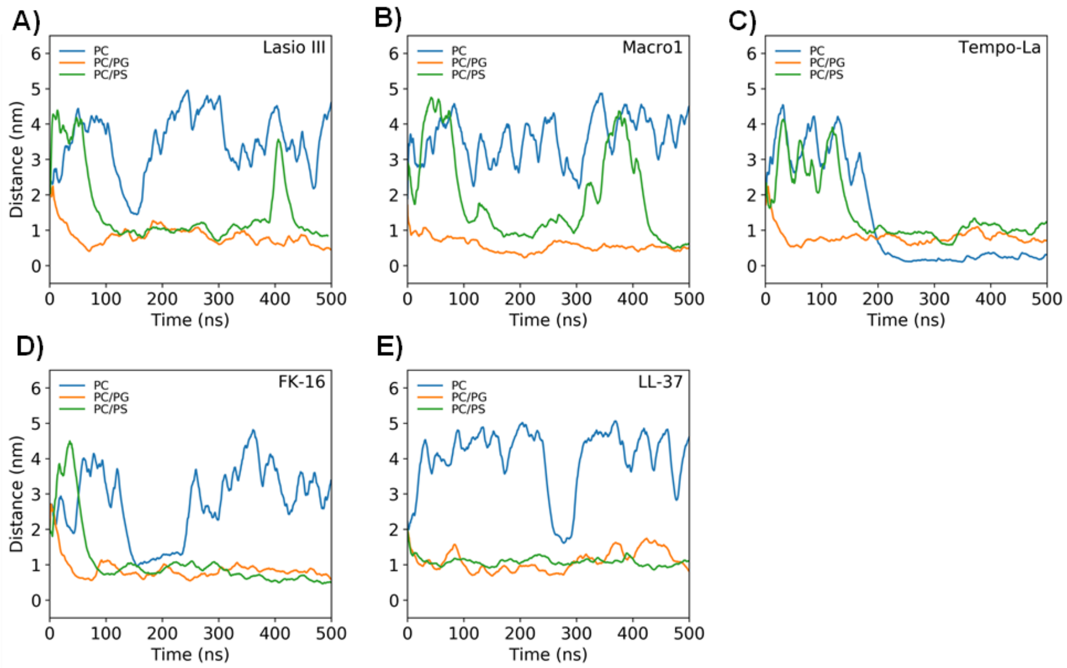

Figure S5. Distance of the peptides from the surface of the different bilayers. The distance is presented as a function of the simulation time and has been grouped by each anticancer peptide used.

REV-ACP interactions studied by CD and FTIR spectroscopy

As previously reported [10], the CD curve of REVs is characterized by two negative bands at ~225 nm and ~210 nm corresponding to the $n \rightarrow \pi^*$ and $\pi \rightarrow \pi^*$ transitions of the peptide bond chromophores, respectively (Figure S6A-C). Addition of LL-37 enhanced the intensity of both minima while FK-16 and Tempo-La induced intensity gain only for the low wavelength band, emerging more as a separate peak in the presence of the peptides. This suggest an increased helicity; however, it cannot be assigned to the ACPs or REV proteins.

Structural effects were further analyzed with IR spectroscopy (Figure S6D-F). The amide I band of REVs is characterized by a main peak centered at 1660 cm^{-1}, indicating the prevalence of α-helical/disordered conformations, and a shoulder at ~1684 cm^{-1} assigned to the β-sheet fraction. No significant changes of the spectral profile were detected upon addition of ACPs. Only LL-37 induced a 2 cm^{-1} downshift of the main peak as well as a moderate shift of the amide II band, indicating an increase of the α-helical fraction upon interaction with the peptide.

The average size was checked using DLS. REVs have a mean diameter of ~200 nm [10]. No significant differences in size and polydispersity were observed after addition of peptides (Figure S3B and Table S1), which indicates that these peptides do not affect the overall integrity of REV vesicles significantly.

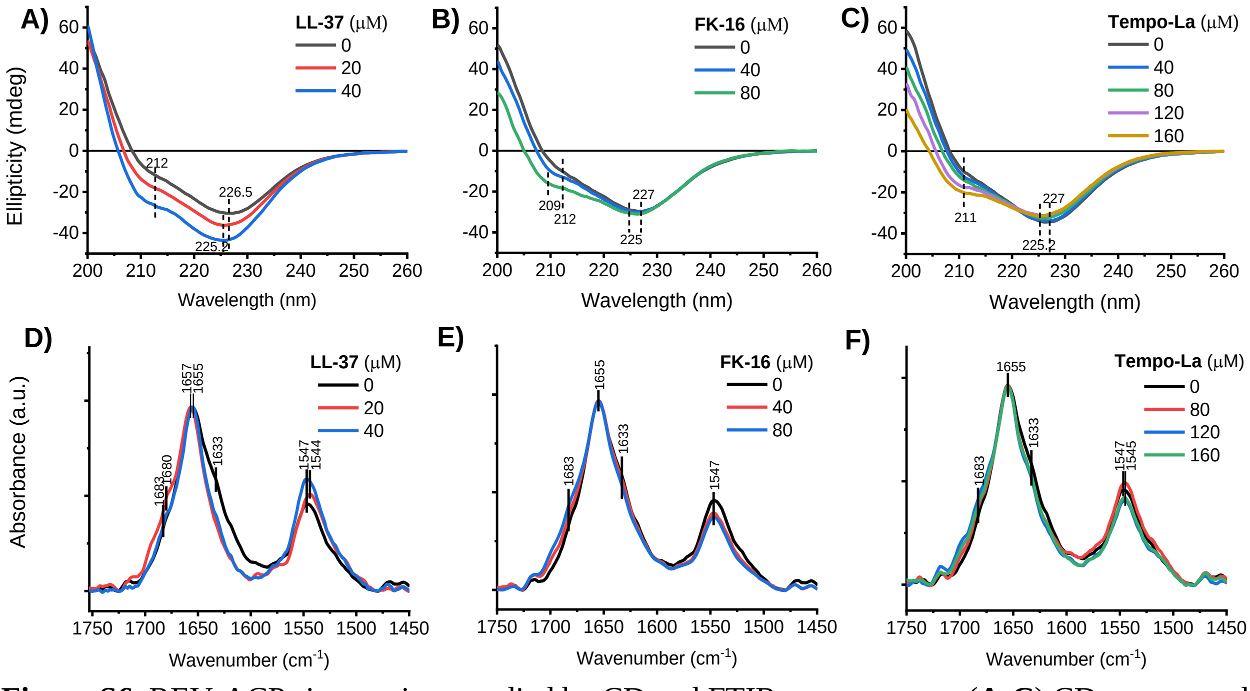

Figure S6. REV-ACPs interactions studied by CD and FTIR spectroscopy. (**A-C**) CD spectra and (**D-F**) infrared spectra of REVs upon stepwise addition LL-37, FK-16 and Tempo-La.

References:

1. Mihály, J.; Deák, R.; Szigyártó, I.C.; Bóta, A.; Beke-Somfai, T.; Varga, Z. Characterization of extracellular vesicles by IR spectroscopy: fast and simple classification based on amide and CH stretching vibrations. *Biochimica et Biophysica Acta (BBA)-Biomembranes* **2017**, *1859*, 459-466.
2. Deák, R.; Mihály, J.; Szigyártó, I.C.; Beke-Somfai, T.; Turiák, L.; Drahos, L.; Wacha, A.; Bóta, A.; Varga, Z. Nanoerythrosomes tailoring: Lipid induced protein scaffolding in ghost membrane derived vesicles. *Materials Science and Engineering: C* **2020**, *109*, 110428.
3. Kranz, J.; AlAzzam, F.; Saluja, A.; Svitel, J.; Al-Azzam, W. Techniques for higher-order structure determination. In *Biophysics for therapeutic protein development*; Springer: 2013; pp. 33-82.
4. Barth, A. Infrared spectroscopy of proteins. *Biochimica et Biophysica Acta (BBA)-Bioenergetics* **2007**, *1767*, 1073-1101.
5. Ismail, A.A.; Mantsch, H.H.; Wong, P.T. Aggregation of chymotrypsinogen: portrait by infrared spectroscopy. *Biochimica et Biophysica Acta (BBA)-Protein Structure and Molecular Enzymology* **1992**, *1121*, 183-188.
6. Dong, A.; Huang, P.; Caughey, W.S. Protein secondary structures in water from second-derivative amide I infrared spectra. *Biochemistry* **1990**, *29*, 3303-3308.
7. Brauner, J.W.; Mendelsohn, R.; Prendergast, F.G. Attenuated total reflectance Fourier transform infrared studies of the interaction of melittin, two fragments of melittin, and. delta.-hemolysin with phosphatidylcholines. *Biochemistry* **1987**, *26*, 8151-8158.
8. Quemé-Peña, M.; Juhász, T.; Mihály, J.; Szigyártó, I.C.; Horváti, K.; Bősze, S.; Henczkó, J.; Pályi, B.; Németh, C.; Varga, Z. Manipulating Active Structure and Function of Cationic Antimicrobial Peptide CM15 by the Polysulfonated Drug Suramin: A Step Closer to in vivo Complexity. *ChemBioChem* **2019**.
9. Dong, A.; Matsuura, J.; Manning, M.C.; Carpenter, J.F. Intermolecular β-sheet results from trifluoroethanol-induced nonnative α-helical structure in β-sheet predominant proteins: infrared and circular dichroism spectroscopic study. *Archives of biochemistry and biophysics* **1998**, *355*, 275-281.
10. Szigyártó, I.C.; Deák, R.; Mihály, J.; Rocha, S.; Zsila, F.; Varga, Z.; Beke-Somfai, T. Flow Alignment of Extracellular Vesicles: Structure and Orientation of Membrane-Associated Bio-macromolecules Studied with Polarized Light. *ChemBioChem* **2018**, *19*, 545-551.
